# An integrated microbiological analysis of the soil and rhizosphere of Agave spp. under minimum technological input farming systems

**DOI:** 10.1099/mic.0.001681

**Published:** 2026-05-15

**Authors:** Victor Henrique Pereira, Gabrielle Henriquetto Cassiano, Pedro Henrique Narciso Ferreira, Luís Guilherme Furlan de Abreu, Juliet Emilia Santos de Sousa, Marcelo Falsarella Carazzolle, Gonçalo Amarante Guimarães Pereira, Nicholas Vinícius Silva

**Affiliations:** 1 Genomics and Bioenergy Laboratory (LGE), Department of Genetics, Evolution, Microbiology and Immunology, Institute of Biology (IB), State University of Campinas (UNICAMP), São Paulo 13083-970, Brazil; 2 Department of Soil Science, Luiz de Queiroz College of Agriculture (ESALQ), University of São Paulo (USP) - Piracicaba, São Paulo 13418-900, Brazil; 3 Center for Computing in Engineering and Sciences, State University of Campinas (UNICAMP), São Paulo 13083-861, Brazil

**Keywords:** *Agave*, enzymatic stoichiometry, micro-organism quantification, soil health, semi-arid, vector analysis

## Abstract

The expansion of arid and semi-arid regions, consequent to the intensification of desertification processes attributable to global warming, exerts a deleterious effect on the agricultural production of energy crops, with current estimates indicating that a further 23% of global agricultural areas will suffer from desertification by 2100, precipitating crises in these sectors. *Agave* species have the capacity to thrive in these marginal environments characterized by aridity and elevated temperatures. These plants can serve as a source of biomass for the production of biofuels, a process that mitigates the environmental impacts of the transport sector while promoting the utilization of drylands, thereby eliminating competition with food crops. Given the paucity of knowledge regarding the soil microbiota and rhizosphere in minimal technological input *Agave* plantations, the objective of this study was to evaluate the microbiological and chemical soil properties of *Agave sisalana* and *Agave* hybrids (H11648 and H400f) farming systems. The analyses, which were carried out using microbial quantification, enzyme stoichiometry and enzymatic vector calculations, demonstrated that the microbiome of these plants is active and well-structured in terms of nutritional acquisition. It was observed that the *Agave* fields’ microbiome is very similar to that of the native vegetation. This finding suggests that the soil and rhizosphere microbiota are healthy and stable in the *Agave* fields evaluated, even with the implementation of agronomic exploitation models, as the chemical analysis of the soil reveals that all measured parameters are consistent with those of soils suitable for crop production. These observations persist even in long-established *Agave* plantations of varying ages that have never received any type of implement or soil correction. Thus, the integration of the chemical and biological data through principal component analysis, redundancy analysis and Permutational Multivariate Analysis of Variance (PERMANOVA) enabled the differentiation of the soil among the three *Agave* species, which shows the influence of the plant genotype on its microbiota.

**Impact Statement**

This article provides new insights into the understudied soil and rhizosphere microbiota of *Agave* plantations in Brazil, to identify points of interest and promote the adoption of this crop for ethanol production in regions with climates unsuitable for planting less resilient varieties, generating savings and social empowerment in vulnerable regions. The data presented suggest that, in the evaluated sites, *Agave* plantations with low technological inputs, even without soil management or correction, appear to maintain soil fertility indicators and high microbial numbers even after prolonged cultivation, an observation not previously reported. The integrated analysis of chemical data, enzyme activity and other soil biological parameters provides information on how different *Agave* genotypes influence the microbiota with which they interact, leading to new studies in this area of research. The correlation between prokaryote quantification by epifluorescence microscopy and soil chemical and biological variables suggests that this method can be used to estimate qualitative soil parameters, which is a novelty in this field of study, as the correlations between microbiological quantification and enzymatic activity remain poorly understood.

## Data Summary

The authors confirm that all supporting data, code and protocols have been provided within the article or through supplementary data files.

## Introduction

Global warming is a current reality; recent models predict that global surface temperatures could rise as much as 4.8 °C by 2100 if sustainable practices are not adopted in production systems, leading to the complete degradation of 12.6% of drylands through aridification and raising 23% of arable land to high-risk categories for desertification [1,2]. These concerns about climate change have inspired global efforts to reduce fossil fuel consumption and greenhouse gas (GHG) emissions [3]. One of the greatest sources of GHG emissions is the transportation sector, which accounts for about 60% of the world’s total oil demand, making it necessary to look for sustainable alternatives [4]. Among the available options, the use of biofuels, such as ethanol, is promising for mitigating the environmental impact of this sector [3,5].

Due to the effects of global warming, there has been an expansion of agricultural areas with arid and semi-arid climates, intensifying natural desertification processes [6]. This has led to the loss of fields previously dedicated to ethanol production, such as sugarcane or maize plantations, making it imperative to identify cultivars tolerant to drought and heat that can meet the global energy demand [7,8]. According to this framework, plants that exhibit superior efficiency in their water usage are particularly notable, especially those classified as having crassulacean acid metabolism (CAM), as these species demonstrate a more effective capacity than C3 and C4 plants in capturing water resources for the purpose of converting CO_2_ into chemical energy through the process of photosynthesis [9].

In this scenario, the CAM plant genus *Agave* (family Asparagaceae, subfamily Agavoideae) is highlighted as one of the most promising biomass sources for the sustainable production of biofuels in regions with climates unfavourable for most of the usual crops, as an alternative to alleviate the world energy crisis with minimal impact and competition for land intended for human food [10,13]. With a focus on the development of *Agave* cultivation, many studies are advancing the exploration of plant–micro-organism relationships and the ecological impacts that large-scale *Agave* cultivation can have on native soil microbiota, especially in Mexico, where *Agave* cultivation is primarily used for tequila production, an alcoholic beverage made from *Agave* distillates [14,17].

Due to restrictions imposed by the Mexican government, a single variety of *Agave* (*Agave tequilana* Weber var. *azul*) can be used in the production of tequila, which causes declines in microbial diversity and impacts the soil in these regions [15,16, 18]. This phenomenon has been documented in metataxonomic studies, which have revealed substantial variations in the alpha diversity of epiphytic and endophytic prokaryotes within monocultures of *A. tequilana* compared to the microbiome of native *Agave salmiana* and *Agave deserti* plants, which is associated with agronomic practices implemented in the tequila industry, such as vegetative reproduction and sterilization of seedling roots before transplanting to the field [19]. These practices favour the enrichment of bacteria belonging to the disease-causing order *Enterobacteriales* in *A. tequilana*, which, in conjunction with the order *Pseudomonadales*, constitute ~92% of the abundance of prokaryotes in a single rhizosphere sample of *A. tequilana* [17].

The microbiota of *Agave* plantations in Brazil remains a relatively understudied area, given the country’s adoption of a natural fibre production model characterized by minimal technological inputs. This approach stands in contrast to Mexico’s intensive cultivation model, which is centred on the large-scale production of ethanol for the tequila industry [20,23]. In Brazil, there are no restrictions on the varieties of *Agave* that can be introduced for fibre production, and three species are commonly used: *Agave sisalana*, *Agave* hybrid H11648 ((*Agave amaniensis* × *Agave angustifolia*) × *A. amaniensis*) and *Agave* hybrid H400f (*Agave* sp., with unknown parents) [24,28]. However, only 3–5% of the biomass is used for fibre extraction, while the remaining material is discarded, despite having significant potential for biofuel production [29].

The greater genetic variability of *Agave* species utilized for fibre production in Brazil may counterbalance the low microbial diversity observed in restricted *A. tequilana* plantations in Mexico, such that the microbiota of these soils may reveal new insights into beneficial plant–micro-organism relationships [23,30, 31]. A technique not yet used to investigate the microbiome and soil health in *Agave* fields in Brazil is the assessment of soil enzymatic stoichiometry. Enzyme stoichiometry is a branch of microbial ecology that provides information about the quality and level of stress to which natural microbial populations are subjected [32,33]. The idea is that the relationship between enzymes involved in the carbon cycle, such as *β*-glucosidases, cellulases, xylanases and dehydrogenases, and enzymes involved in the nitrogen (ureases and proteases) and phosphorus (phosphatases) cycles indicates the nutritional limitations imposed on micro-organisms under the influence of abiotic and biotic factors, based on the creation and analysis of vectors that integrate these three classes of enzymes, called ecoenzymes (EEA) because of how they affect nutrient cycling [32,34,39].

The association between enzyme stoichiometry and microbial quantification, combined with soil chemical parameters, can provide new insights into the studied microbiota, with calculations of correlations between microbial quantification and enzyme activity, and microbial coefficients between elemental and energetic flows of microbiota with the environment [40,44]. Therefore, given the recent importance of heat-tolerant vegetable crops, the present study aims to evaluate the soil indicators and rhizosphere microbiota of *Agave* fields in the semi-arid region of Brazil. For this purpose, three *Agave* species (*A. sisalana*, H11648 and H400f) were selected for the study in very close productive areas, but with differences in planting type and age. In this way, we were able to assess how different management practices and *Agave* species affect the soil and rhizosphere microbiota of these fields, with large cultivation windows that allow us to distinguish how microbiological populations are modified over time in interaction with the *Agave* plants. Stoichiometric and enzyme vector calculations are employed to indicate the nutritional relationship between microbial biomass and the availability of macronutrients (carbon, nitrogen and phosphorus) in these soils. The proportions between exudates of EEA in the environment are indicative of the tendency to acquire a limiting nutrient, a principle that is supported by the chemical soil assessment [32,45, 46]. Therefore, the study of these enzymatic parameters will be utilized to estimate soil microbiological and chemical conditions in *Agave* spp. plantations.

## Methods

### Sampling

**Fig. 1.**
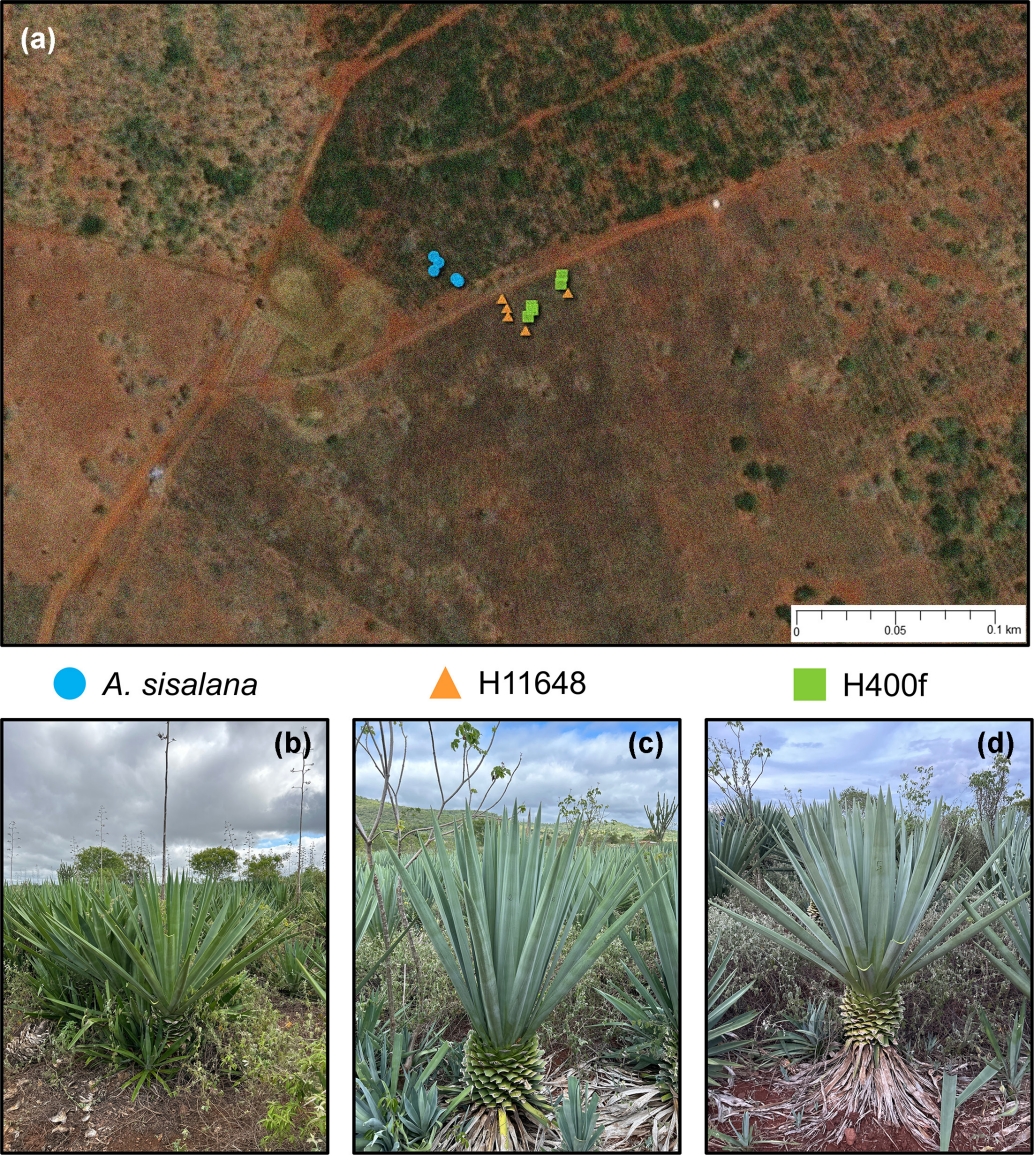
Soil and rhizosphere sampling of *A. sisalana*, H11648 and H400f: (a) region where sampling was carried out (image: ArcGIS Online v.2.8); (b) *A. sisalana*; (c) H11648; (d) H400f.

Samples were collected in the semi-arid region of Brazil (11° 10′ 07.3″ S 39° 17′ 10.4″W) (Fig. 1a), in an area with an elevation of 390 m, a mean annual precipitation of 33 mm and a stable mean annual temperature between 20 and 34 °C throughout the day. Three species of *Agave* were chosen for sampling: (i) *A. sisalana,* (ii) H11649 and (iii) H400f (Fig. 1b–d). This region was selected for collection because it contains, in a small geographical space, the three *Agave* species planted in soils of the same classification; however, the *A. sisalana* plantation is over 40 years old, where the plants are arranged in random pits, without any type of agricultural management carried out throughout the cultivation period. The hybrids, on the other hand, were planted in 2019 side by side in a row scheme, interspersed with H11648 and H400f, but also without any type of fertilization or soil management. These plants are used for the production of sisal fibre, with the leaves cut by hand every 2 years. The chosen area had many of the characteristics we needed to conduct the study, such as different planting times and schemes, with different *Agave* genotypes planted in relatively similar soil. However, this study is limited to the area in question; further studies in other regions with *Agave* plantations are needed to confirm the results presented in this article.

Soil was collected at depths between 0 and 20 cm by combining three sampling points in a triangular pattern around the plant, up to 20 cm from the plant of origin. After collection, the soil was analysed for microbial quantification, chemical analysis and enzymatic activity. The rhizosphere was sampled from the secondary roots of the same plants from which the soil was collected. The collected rhizosphere was used only for microbiological quantification assessments. A total of 5 plants were sampled for each *Agave* species (5 biological replicates), for a total of 30 samples (15 soil and 15 rhizosphere). All samples were stored at −20 °C until use [47,48].

## Micro-organism quantification

To ensure accurate microbial quantification, four methods were applied to soil and rhizosphere samples [49]: (i) c.f.u. counting, using tryptic soy agar (TSA) culture media to count cultivable prokaryotes and dichloran rose bengal chloramphenicol (DRBC) to quantify filamentous fungi and yeasts [50] (Fig. S1, available in the online Supplementary Material); (ii) most probable number (MPN), using tryptic soy broth medium for microbial cultivation and iodonitrotetrazolium chloride (INT) to confirm microbial growth [51] (Fig. S2); (iii) total DNA extraction (TDE), using *QIAGEN*^®^ *DNeasy PowerSoil Pro Kit* for DNA extraction and quantification by spectrophotometry and fluorimetry [52]; and (iv) epifluorescence microscopy (EPI), using the *LIVE/DEAD*^™^ *BacLight*^™^ kit to label viable and non-viable cells, and visualization of images on a Zeiss-Apotome3^™^ fluorescence microscope, from the average cell count performed on a mosaic of 25 landscapes, analysed using *ImageJ-Fiji v.1.54* software [53] (Fig. S3).

The expected results for the four microbiological enumeration methods are as follows: (i) c.f.u.: a culture-dependent method involving the direct counting of colonies in general culture media for prokaryotes and fungi; (ii) MPN: a culture-dependent method that estimates the number of culturable micro-organisms. Chemical markers were used to count specific microbial subsets, e.g. INT reduction is used to quantify aerobic micro-organisms; (iii) TDE: a culture-independent method that extracts total genetic material from soil and calculates the number of cells using an average amount of DNA per cell; however, this method may overestimate results due to the extraction of non-microbial DNA; (iv) EPI: culture-independent method that involves the utilization of microscopic counting using fluorescent markers that stain live and dead cells in solution, this method is used to achieve a higher resolution of the actual amount of live micro-organisms in the soil.

## Soil chemical analysis

Soil chemical analyses were conducted according to Jackson [54], Juo et al. [55], Mclean [56], Black [57], van Raiji [58] and Mehlich [59], with samples obtained by combining five soil samples for each *Agave* species (sample pool). The pH was measured in water and in potassium chloride (KCl) (1 mol.l^−1^), the potential acidity [PA − (H^+^ + Al^3+^)] by extraction with calcium acetate (1 mol.l^−1^) and determination by titration. Calcium (Ca) and magnesium (Mg) were quantified with KCl (1 mol.l^−1^) and determined with an atomic absorption spectrophotometer. Aluminium (Al) was extracted with KCl (1 mol.l^−1^) and determined by titrimetry. Potassium (K) and sodium (Na) were extracted using the Mehlich 1 method and quantified by flame photometry. Phosphorus (P) extraction with Mehlich 1 and determination by colourimetry. Determination of organic matter (OM) by oxy-reduction titration using K_2_Cr_2_O_7_. Total nitrogen (TN) was determined through extraction, Kjeldahl distillation and titration using acid–base indicators. Sum of bases (SB), cation exchange capacity (CEC), base saturation (V%), aluminium saturation (m%) and total organic carbon (TOC) were calculated from the following data.

## Evaluation of the enzymatic activity of EEA

The enzymatic activities of the enzymes *β*-glucosidase, arylsulphatase and acid and alkaline phosphatases were carried out according to Alef and Nannipieri [60], evaluated by colourimetry based on the release of P-nitrophenol following enzymatic hydrolysis in buffer solutions specific to each enzyme. The activities of cellulase and xylanase were evaluated in a colourimetric assay based on the reduction of 3,5-dinitrosalicylic acid caused by the release of reducing sugars into solution after the degradation of cellulose (Avicell) and xylan polymers [61]. Protease activity was assessed according to Jesmin *et al.* [62], with a colourimetric assay carried out by reducing the Folin–Ciocalteu reagent, where tyrosine, released by the hydrolysis of sodium caseinate mediated by proteases, acts as the reducing agent. Urease enzyme activity was measured by the Kjeldahl method using the evolution of ammonia and ammonium from urea, followed by titration of the distillate with acid–base indicators [60]. Dehydrogenase activity was evaluated according to Suliman *et al.* [63], with colourimetric evaluation of the formation of triphenyltetrazolium formazan (TTF) from triphenyltetrazolium chloride salt.

## Soil microbiological parameters

Microbial biomass carbon (MBC) and microbial biomass nitrogen (MBN) were determined using the fumigation–extraction method, followed by quantification by titration with potassium dichromate and ferroin indicator for carbon, and distillation (Kjeldahl) and titration with acid–base indicators for nitrogen [64,65]. Basal microbial respiration (MR) was assessed by trapping CO_2_ in a NaOH solution (0.1 mol.l^−1^), followed by titration with phenolphthalein as an acid–base indicator [54].

The metabolic quotient (*q*CO_2_) was calculated from the ratio between soil basal MR in mg CO_2_.g soil^−1^.h^−1^ and MBC in g C.g soil^−1^, while the microbial quotient (C_mic_/C_org_) was calculated from the ratio between MBC and TOC in standardized values in µmol for TOC and MBC per gram of soil [40].

## Data analysis and statistics

Statistical analyses were performed using *GraphPad Prism v.9.0.0*, *R v.4.5.0* and *Sisvar v.5.3*, with statistical significance set at *P*<0.05. All individual results were subjected to the Shapiro–Wilk test for normality, followed by ANOVA and Tukey’s test for multiple comparisons. After data standardization (z-score), principal component analysis (PCA) was performed on 25 variables (excluding quantification data due to low statistical difference between samples, with the first and second principal components tested for normality using the Shapiro–Wilk test (Fig. S4), ANOVA for analysis of variance, Tukey’s test for multiple comparisons, Pearson’s correlation and clustering (*k*=3) (Fig. S1). Since some variables did not follow a normal pattern of dispersion, the Levene, Kruskal–Wallis and Dunn tests were used to confirm the results obtained (Table S1), confirming that non-parametric tests were not required in this context. To include the quantification data in the analysis, the Shapiro–Wilk normality test was performed, and the Spearman (non-parametric) correlation matrix was calculated, with the results expressed as correlation indices and *P*-value. We performed redundancy analysis (RDA) (Fig. 7) and incorporated the environmental variables TOC and TN into the model following selection based on a variance inflation factor (VIF) of less than 2. The global significance and that of the individual axes and terms of the model were assessed with 999 permutations. Permutational Multivariate Analysis of Variance (PERMANOVA) was performed to identify differences between the groups of *Agave* species evaluated (Fig. S8). The distance matrix was calculated using the Euclidean metric with 999 permutations, and a multivariate dispersion test was performed to confirm homogeneous dispersion, thereby validating the results obtained.

## Results and discussion

### Micro-organism quantification

**Fig. 2.**
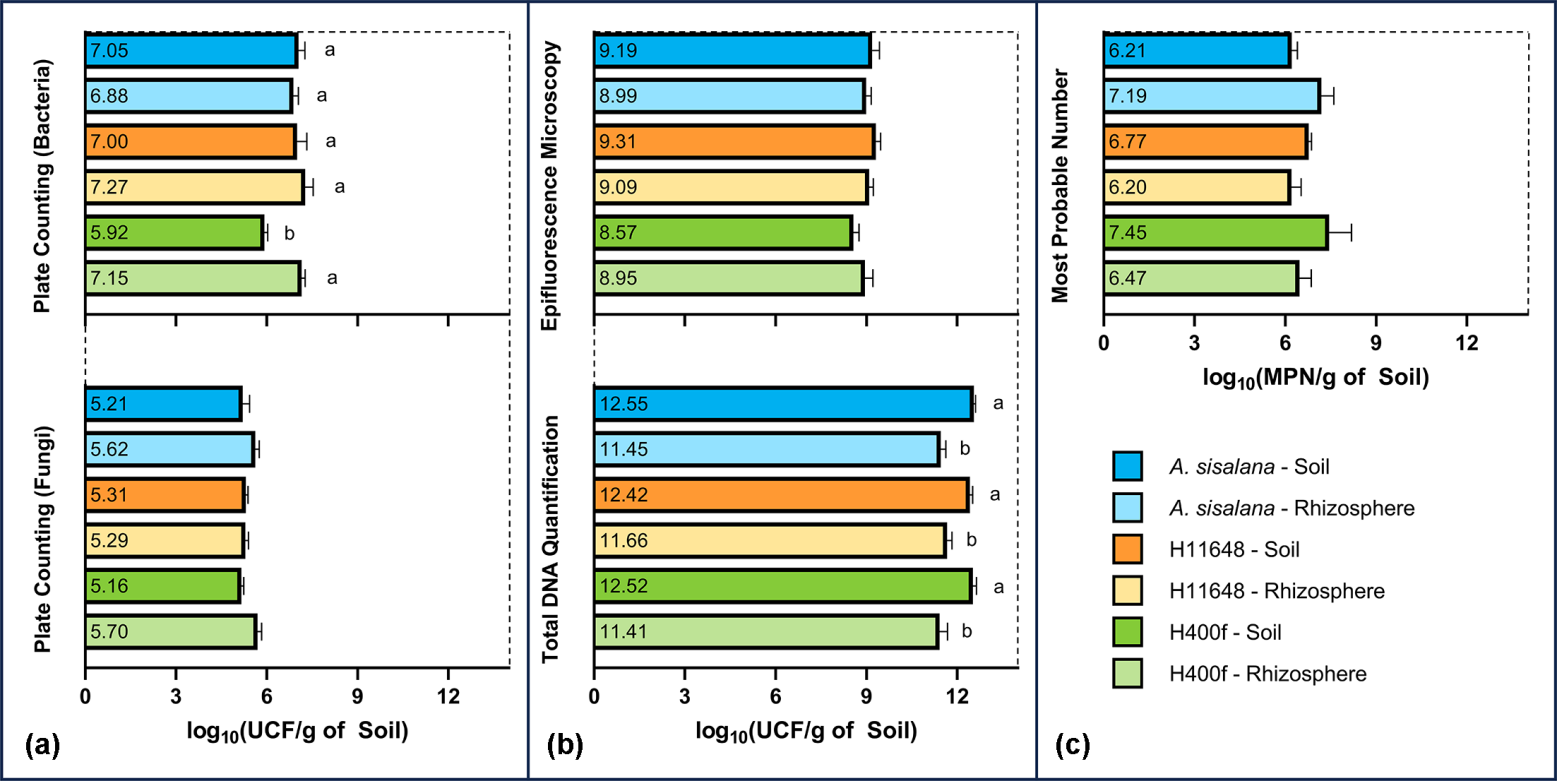
Quantifications of micro-organisms for soil and rhizosphere of *A. sisalana*, H11648 and H400f. (a) c.f.u. method for bacteria (above) and fungi (below). (b) Quantification method by EPI (above) and quantification by TDE (below). (c) Quantification method by MPN.

The number of micro-organisms in the samples varied depending on the method employed (Fig. 2) (Table S1). The c.f.u. method utilizes generalized media to count prokaryotes (TSA medium) and fungi (DRBC medium), counting the cultivable micro-organisms present in the soil and rhizosphere [50] (Fig. 2a). The MPN technique (Fig. 2c) relies on statistical inference to estimate the abundance of culturable micro-organisms and generally offers higher sensitivity than the c.f.u. method [49]. However, MPN relies on the reduction of the INT reagent by aerobic micro-organisms to iodonitrotetrazolium violet-formazan, a process mediated by dehydrogenases, especially the NADH dehydrogenase located in mitochondria or their prokaryotic analogues [51,66, 67].

To enumerate the ‘non-culturable’ micro-organisms in the soil and rhizosphere, the culture-independent methods (Fig. 2b) included the TDE method, in which DNA was extracted from the soil and rhizosphere using commercial kits, and the number of cells was counted using 8.14 fg (10^−15^ g) of DNA per bacterial cell as the base value [52]. Another culture-independent method used was EPI, which uses the fluorescent markers propidium iodide (red fluorescence) and SYTO-9 (green fluorescence) to label viable and non-viable cells in solution. The number of cells is then determined through microscopy imaging and image analysis software [53,68]. This method provides the highest resolution for estimating the actual microbial abundance in soil and rhizosphere, including both culturable and ‘non-culturable’ organisms [49].

The data obtained from these quantification approaches revealed that few significant differences were found for the soil and rhizosphere of the three *Agave* species, with the only notable distinction being a lower c.f.u. count for bacteria in the soil of H400f. This result suggests that the microbial communities across these species are relatively balanced, despite the differences in management and planting time, particularly between *A. sisalana* and the hybrids, a pattern also reported in previous studies combining metataxonomics and microbial quantification across geographically distant *Agave* populations [17,23, 69, 70]. The only test that consistently detected a difference between soil and rhizosphere was TDE, which may be an artefact of nucleic acid extraction for rhizosphere, which generally yields less DNA than soil extractions due to sample preparation requirements in commercial kits [48]. Despite this, the overall similarity in microbial abundance between soil and rhizosphere may suggest that *Agave* exhibits selectivity in recruiting micro-organisms via root exudates, considering that the rhizosphere typically contains more nutrients and supports a higher density of microbial cells [25,71, 72].

The similarity between c.f.u. and MPN counts indicates that the community of culturable micro-organisms in these samples comprises predominantly aerobic representatives, which is an indication of accelerated microbial metabolism involved in the decomposition of organic matter in these environments [73,74], reflecting an active carbon cycle [75]. The higher values observed through TDE compared to other methods have already been reported in previous studies [49]. This discrepancy is attributed to the inability to separate DNA from living and dead cells during the nucleic acid extraction process, which also results in the extraction of plant and animal DNA dispersed in the samples along with microbial DNA, leading to an overestimation of microbial abundance [52]. In contrast, EPI counting allows a more accurate estimation of viable micro-organisms, addressing the so-called great plate count anomaly, which distinguishes between culturable and ‘non-culturable’ micro-organisms [76,77].

The mean total microbial counts (10^7^ c.f.u.g soil^−1^ for culturable and 10^9^ c.f.u.g soil^−1^ for ‘non-culturable’) are comparable to those reported for soils from caatinga biome (native dry forest in the region) and other natural forests [78,81]. These values are higher than those normally found in soils under conventional agricultural systems, 10^6^ c.f.u.g soil^−1^ for culturable and 10^8^ c.f.u.g soil^−1^ for ‘non-culturable’ [82,83]. The intensive cultivation and the continuous removal of plant biomass over the years can deplete soil resources, thereby negatively affecting the microbiota and reducing the abundance of micro-organisms in the soil [84]. Within the conditions evaluated, no reduction in the number of micro-organisms in the soil solution was observed in *Agave* plantations, even considering their longevity, the absence of fertilization and the constant removal of biomass.

We hypothesize that *Agave* plants have strategies for maintaining active and stable soil microbiota. In *Agave lechuguilla*, root exudates recruit beneficial rhizobacteria, primarily through the production of amino acids, such as tyrosine and arginine [85]. Metataxonomic studies of *A. lechuguilla* revealed that the rhizosphere community differs from the one found in bulk soil, where the rhizosphere possesses a greater presence of microbial species with functional traits that promote plant health and growth [69]. Other metataxonomic studies also suggest that *Agave* resistance to adverse edaphoclimatic conditions is associated with the microbial communities these plants harbours, serving as a host for numerous species of microbes capable of positive interspecific interactions, ensuring the plant’s survival in severe climates [17,19, 69, 70, 85].

## Soil chemical analysis

The results of the soil chemical analysis are presented in Table 1. The pH values in the water for the three *Agave* species were greater than 6.5, which is considered a slightly acidic soil [86], rendering insoluble forms of aluminium that are toxic to plants, which explains the results found for Al^3+^ and m%. The values found for OM are greater than 30 g.kg^−1^ for the three *Agave* species, which are considered soils rich in organic matter [87], with lower values for the *A. sisalana* soil compared to the hybrids. The elevated concentrations of essential cations (Ca²^+^, K^+^, Mg²^+^ and Na^+^) observed in the soils cultivated with the hybrid *Agave* species, compared to those cultivated with *A. sisalana*, may be partially explained by the greater OM content in the hybrid’s soils, given the ability of OM to retain cations [88].

**Table 1.** Chemical analysis of the bulk soil of *A. sisalana*, H11648 and H400f

Soil parameter		*A. sisalana*	H11648	H400f
pH – H_2_O	**–**	6.67	6.71	6.9
pH – KCl	**–**	5.36	5.6	5.84
OM	g.kg^−1^	37.5	56.6	54.4
TOC	g.kg^−1^	21.75	32.83	31.55
TN	mg.kg^−1^	2,905	3,290	1,918
P	mg.kg^−1^	12.2	44	32.5
CS/PS	**–**	4,597.2	1,923.9	2,503.5
CS/NS	**–**	8.7	11.6	19.2
NS/PS	**–**	526.7	165.4	130.6
Ca	mmol_c_.kg^−1^	269.5	292.1	279.1
K	mmol_c_.kg^−1^	3.4	3.8	5.2
Mg	mmol_c_.kg^−1^	142.1	110.3	118.5
NA	mmol_c_.kg^−1^	6.3	4.1	4.3
Al	mmol_c_.kg^−1^	0	0	0
PA	mmol_c_.kg^−1^	34.8	41.2	12.8
SB	mmol_c_.kg^−1^	421	410	407
CEC	mmol_c_.kg^−1^	456	452	420
V%	%	92	91	97
m%	%	0	0	0

Al, Aluminum; Ca, Calcium; CS/NS, TOC/TN [(µmolC.g soil-1)/(µmolN.g soil-1)]; CS/PS, TOC/P [(µmolC.g soil-1)/(µmolP.g soil-1)]; K, Potassium; Mg, Magnesium; Na, Sodium; NS/PS, [(µmolN.g soil-1)/(µmolP.g soil-1)]; P, Phosphorus; PA, Potencial Acidity.

The OM levels were used to estimate TOC in the soil samples, following the methodology described by Findorákova *et al.* [89]. The TOC content exceeded 30 g.kg^−1^ in soils cultivated with hybrids and 20 g.kg^−1^ in soils with *A. sisalana*. According to Chen *et al.* [90], TOC values above 20 g.kg^−1^ are considered high, corroborating the previously observed OM levels in these soils. TN concentrations varied among the *Agave* species, with the lowest value recorded for the hybrid H400f (1,918 mg.kg^−1^) and the highest for *A. sisalana* and H11648 (>2,500 mg.kg^−1^). All TN values fall within the optimal range for plant development, indicating neither nitrogen limitation nor excessive nitrogen accumulation [91,92].

The high levels of essential cations (SB >400 mmol_c_.kg^−1^) and medium levels of PA (25–50 mmol_c_.kg^−1^, except for H400f) in these soils are critical factors in the CEC and V% calculations and demonstrate the buffering nature of these soils. However, the pH is already close to optimal range for plant cultivation (6.5) [93,94], and the elevated concentrations of essential cations (SB) relative to H^+^ and Al^3+^ (PA) raise the V% in these soils to values above 90%, which, together with the phosphorus levels (>10 mg.kg^−1^), indicate that these soils are suitable for the cultivation of most crops [95,96]. Notably, these conditions were observed despite the prolonged cultivation of *Agave* in the region without the application of fertilizers or soil amendments, indicating a gradual preservation of mineral elements over successive years of agronomic exploitation [97,98].

The OM and TOC contents in the soil are essential for the production of hydrolases by soil micro-organisms for nutritional acquisition, e.g. cellulases, ureases and arylsulphatases[99]. Low carbon levels severely limit microbial activity in these environments [100]. Additionally, OM can form humic complexes that slow the degradation of EEAs, thereby maintaining the enzymatic stability in the soil [101]. Other growth-limiting elements are nitrogen and phosphorus, and the nutritional limitation of these elements is assessed by the production of EEAs for the uptake of these nutrients [102,103]. An inverse relationship is often observed between the production of enzymes for nutritional uptake and the availability of the relevant element in the soil, e.g. phosphatases and P availability in the soil [104]. However, this is not a fundamental law, as distinct cases have already been observed [32].

The ionic composition of soil significantly influences microbial activity and is directly associated with soil pH [105]. Acidic soils tend to release aluminium, which can impair the activity of enzymes that are sensitive to this element [106]. Basic cations, such as Ca²^+^ and Mg²^+^, improve soil structure and act as essential nutrients for microbial metabolism and EEA’s stability [107,108]. K^+^ aids microbial development but has a lesser direct influence on enzymatic activity than Ca²^+^ and Mg²^+^ [109]. Excess Na^+^ can cause osmotic stress in microbial cells, reducing the overall metabolic activity in saline environments. Proper Ca:Mg:K:Na ionic balance is essential for microbial metabolism in soil, and pH must be between 5.5 and 7 to maximize essential nutrient availability, while preventing the formation of toxic aluminium compounds [110,111]. Also, this range of pH is close to the optimal for most EEA’s reactions [110].

## Enzymatic activity of ecoenzymes and soil microbiological parameters

EEAs are enzymes exuded by microbial cells in the soil and rhizosphere in response to factors, such as nutrient availability, in order to shape the environment in favour of biological development [32]. The results (Table 2) for EEAs that mineralize carbon in the form of glucose (*β*-glucosidase and cellulases) and xylose (xylanases) demonstrate the capacity of these soils to recycle organic matter through the oxidation of complex carbon polymers, including xylan or cellulose, which represent the primary sources of carbon inputs in natural soils [32]. The combined activity of cellulases (endocellulases, exocellulases and *β*-glucosidase) was 0.135 µmol glucose.g soil^−1^.h^−1^ on average for the three *Agave* species. In contrast, *β*-glucosidase activity alone averaged 0.466 µmol P-nitrophenol.g soil^−1^.h^−1^. Xylanase activity averaged 0.498 µmol glucose.g soil^−1^.h^−1^, a value close to that observed for *β*-glucosidase activity measured under conditions that maximize carbon conversion.

**Table 2.** Results for the enzymatic activity of arylsulphatase, acid and alkaline phosphatase, *β*-glucosidase, cellulase, xylanase, dehydrogenase, protease and urease Enzyme calculation values for carbon (CE), nitrogen (NE) and phosphorus (PE) and their ratios. Length and angle values of enzyme vectors. For *A. sisalana*, H11648, H400f soils and the average between the three *Agave* species.

Enzyme	Activity	*A. sisalana*	H11648	H400f	Mean	
**Arylsulphatase**	µmol P-nitrophenol.g soil^−1^.h^−1^	1.764^ns^	1.936^ns^	2.354^ns^	2.018	
**Alkaline phosphatase**		40.645^ns^	41.570^ns^	44.405^ns^	42.207	
**Acid phosphatase**		27.415^ns^	39.865^ns^	29.308^ns^	32.196	
***β*-Glucosidase**		0.358^ns^	0.491^ns^	0.550^ns^	0.466	
**Cellulase**	µmol glucose.g soil^−1^.h^−1^	0.146^ns^	0.172^ns^	0.086^ns^	0.135	
**Xylanase**		0.391^ns^	0.673^ns^	0.428^ns^	0.498	
**Dehydrogenase**	µmol TTF.g soil^−1^.h^−1^	2.35e−03^ns^	2.44e−2^ns^	7.06e−3^ns^	3.95e-3	
**Protease**	µmol l-tyrosine.g soil^−1^.h^−1^	0.217^ns^	0.673^ns^	0.237^ns^	0.376	
**Urease**	µmol NH_4_^+^.g soil^−1^.h^−1^	10.775^ns^	8.417^ns^	7.191^ns^	8.794	
**Carbon enzymes (CE)**	µmol product.g soil^−1^.h^−1^	0.893^ns^	1.334^ns^	1.057^ns^	1.095	
**Nitrogen enzymes (NE)**		10.992^ns^	9.090^ns^	7.427^ns^	9.170	
**Phosphorus enzymes (PE)**		27.415^ns^	39.865^ns^	29.308^ns^	32.196	
**CE/PE**	–	0.033^ns^	0.031^ns^	0.036^ns^	0.033	
**CE/NE**		0.083^ns^	0.162^ns^	0.149^ns^	0.131	
**NE/PE**		0.405^ns^	0.254^ns^	0.254^ns^	0.304	
**Vector length**		0.083^ns^	0.136^ns^	0.134^ns^	0.118	
**Vector angle**	Degrees (°)	67.231^ns^	74.658^ns^	74.460^ns^	72.116	

NS = not significant (p > 0.05).

NS, Non significative results at 5% significance level.

In addition to carbon-evolving enzymes, dehydrogenase activity was evaluated, primarily that of NADH dehydrogenase, which is associated with the consumption of hexoses and pentoses via aerobic metabolism pathways [36,112]. The mean activity of dehydrogenases was 3.95×10⁻³ µmol TTF.g soil^−1^.h^−1^ across all *Agave* species (Table 2), a value lower than that of carbon-releasing enzymes, which suggests carbon accumulation in the soil, indicating a greater tendency towards the mineralization of this element [113].

For the sulphur cycle, we evaluated the activity of arylsulphatases, which are enzymes capable of releasing free sulphates (SO_4_^2-^) from sulphate esters (R-OSO^3-^) into the soil [114]. The average activity of arylsulphatases for the three *Agave* species was 2.018 µmol P-nitrophenol.g soil^−1^.h^−1^ (Table 2), a value comparable to that found in organically managed systems and higher than in conventionally managed plantations, that typically present activity close to 1.0 µmol P-nitrophenol.g soil^−1^.h^−1^ [115].

The activity of acid phosphatases (pH 6.5) and alkaline phosphatases (pH 8) was also evaluated, with average values of 32.2 and 42.2 µmol P-nitrophenol.g soil^−1^.h^−1^, respectively, across soils from the three *Agave* species (Table 2). These values are substantially higher than those observed in native caatinga soils during the rainy season, e.g. 6.5 µmol P-nitrophenol.g soil^−1^.h^−1^ [116]. However, these numbers are within those observed in global reviews for phosphatase activity, in the highest range of activities evaluated (>40 µmol P-nitrophenol.g soil^−1^.h^−1^) [36]. The inversely proportional relationship between the activity of phosphatases and the amount of P available in the soil is well known; increased activity of these enzymes may indicate P limitation in the soil [117]. Furthermore, the ratio between the average activity of alkaline and acid phosphatases (alkaline phosphatase/acid phosphatase=AlkP/AcdP) can also be used as an indication of fertility and the need for liming in soils [118]. On average, the three *Agave* species studied have AlkP/AcdP=1.3; this value is greater than 0.5, indicating that there is no need to correct these soils for agronomic cultivation [118].

Regarding the nitrogen cycle, the enzymes proteases and ureases were assessed. Proteases are responsible for the degradation of proteins via peptide bond cleavage, while ureases catalyse the release of ammonia and ammonium ions from urea and other amides in soil. The combined activity of these two families of enzymes is commonly used as an indicator of nitrogen availability in the soil, since the main sources of nitrogen in the soil are ammonium ions [39] and plant-derived proteins [38], followed by the cell wall components of filamentous fungi and yeasts (assessed through chitinase activity) [32]. The average enzymatic activity of the three *Agave* species analysed was 0.376 µmol l-tyrosine.g soil^−1^.h^−1^ and 8.794 µmol NH^4+^-N.g soil^−1^.h^−1^ for proteases and ureases, respectively (Table 2). The higher value for urease compared to proteases indicates that the main source of N in these soils is low molecular weight molecules with C-N bonds outside of peptide bonds, mainly in amide bonds [119], as the expression of ureases is positively correlated with the presence of amides in soil, while proteases are correlated with the presence of peptides [32]. Both enzymes are negatively affected by elevated soil ammonium concentrations and the activities measured here suggest that the inhibitory threshold has not yet been reached [32,120, 121].

Despite the variability in enzymatic activity values (Table S1), no statistically significant differences were observed among the three *Agave* species for any of the nine enzymes evaluated (Table 2). However, we observed a tendency for *A. sisalana* soil to have less enzymatic activity compared to the hybrid’s soil (except for urease and cellulase activity). This may reveal a shift in nutrient acquisition metabolism towards cell maintenance and multiplication in the microbiota of these soils, referring to the nutritional stability acquired in the soils after many years of *Agave* cultivation [122]. Therefore, the lower hydrolase activity in *A. sisalana* soils suggests that these soils may be closer to a balanced C/N/P stoichiometry compared to hybrid soils.

This finding indicates a consistent microbiological balance among the soils, with microbial communities seemingly structured to optimize nutrient acquisition, especially in the soil of *Agave* hybrids [123,124]. In general, all EEA showed results above those found in agricultural soils, with values close to those of the native vegetation of the region [116,125,129]. These results demonstrate the ecological quality and functional integrity of soils under *Agave* cultivation, which, despite the absence of agricultural supplementation, maintain nutrient cycling rates and biological activity comparable to those of native ecosystems.

**Fig. 3.**
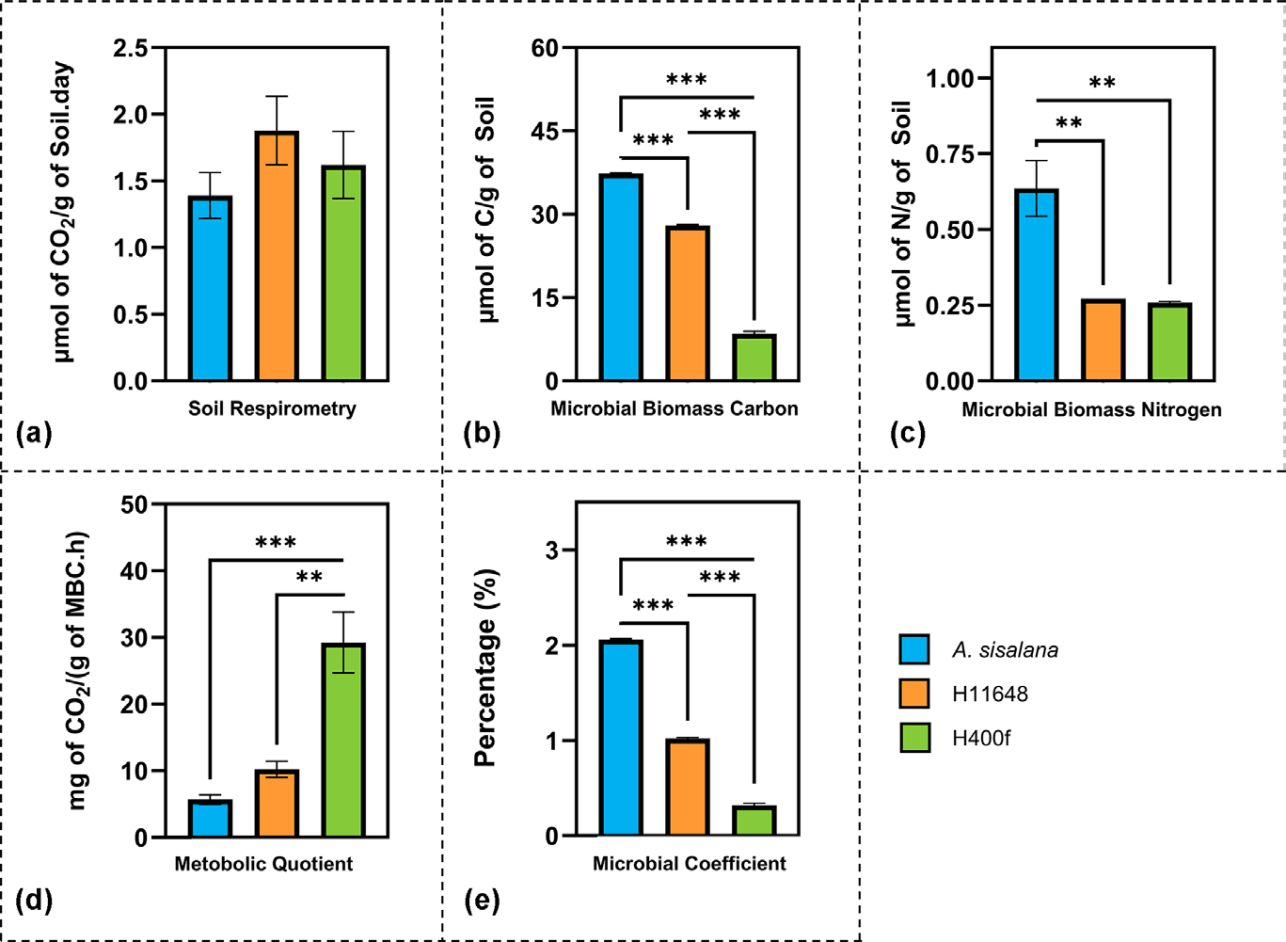
Microbiological parameters of *A. sisalana*, H11648 and H400f soils. (a) Basal MR, (b) MBC, (c) MBN, (d) metabolic quotient (*q*CO_2_); microbial coefficient (C_mic_/C_org_). * The asterisks represent the levels of statistical significance: **p ≤ 0.01, and ***p ≤ 0.001.

In addition to EAAs, microbiological parameters can be assessed to evaluate soil quality and health. The basal MR showed no statistical difference between the *Agave* species (Fig. 3a), with an average value of 1.61 µmol CO_2_.g soil^−1^.day^−1^ for all three *Agave* species. This value is higher than that observed in restored agricultural fields (<10 g CO_2_.m^2^ soil^−1^day^−1^), lower than that observed in degraded steppes (>10 g CO_2_.m^2^ soil^−1^.day^−1^) and similar to the MR of natural steppes (≈15 g CO_2_.m^2^ soil^−1^day^−1^) [130]. The evaluated MR is also twice that of secondary coniferous forest soils in China (≈0.8 µmol CO_2_.g soil^−1^ day^−1^) [131] and three times greater than that of desert regions (≈0.5 µmol CO_2_.g soil^−1^.day^−1^) [40]. The closest MR values to soils for the three *Agave* species studied are those of tropical forests [132], demonstrating the effective capacity of organic matter decomposition in these soils. These comparisons suggest a high capacity for C mineralization in *Agave* soils, which supports the hypothesis that these soils have a greater quantity of aerobic micro-organisms, as evidenced by the differences between the c.f.u. and MPN methods for enumeration of cultivable micro-organisms.

Other microbiological parameters differentiated the soil of the different *Agave* species. We observed significant variations in MBC among *A. sisalana* (37.31 µmol C.g soil^−1^), H11648 (28.0 µmol C.g soil^−1^) and H400f (8.46 µmol C.g soil^−1^) (Fig. 3b). The MBC values for *A. sisalana* and H11648 are similar to those found in tropical and subtropical forests and shrubland soils, while the MBC values for H400f are more similar to those evaluated in desert and cropland soils [126,133]. This difference in MBC values may suggest a greater carbon allocation to microbial growth in the microbial communities associated with *A. sisalana* and H11648 and a greater conversion of carbon to maintenance and stocking of organic molecules in H400f [134,135], demonstrating the capacity of distinct *Agave* genotypes to modulate their associated microbiomes, even under similar soil conditions [69].

The difference between the observed MBC can be explained by the significant differences evaluated in the metabolic quotient (*q*CO_2_) (Fig. 3d) and microbial coefficient (C_mic_/C_org_) (Fig. 3e) among the three *Agave* species. The average observed metabolic quotient (*q*CO_2_) was 15.06 mg CO_2_.g MBC^−1^.h^−1^, and the microbial coefficient (C_mic_/C_org_) was 1.14%, reflecting microbial energy requirements and carbon availability, respectively [40,136].

A higher value for *q*CO_2_ indicates a greater energy allocation to microbial respiration and maintenance rather than to biomass production [137,138]. The highest *q*CO_2_ was observed in H400f (29.24 mg CO_2_.g MBC^−1^.h^−1^), followed by H11648 (10.23 mg CO_2_.g MBC^−1^.h^−1^) and *A. sisalana* (5.69 mg CO_2_.g MBC^−1^.h^−1^), suggesting a relatively greater physiological stress in the microbiota of the hybrids compared to *A. sisalana*. This also implies lower OM quality in hybrid soils, where OM with high lignin content, a very recalcitrant carbon molecule, may be predominant [139,140].

Significant differences in C_mic_/C_org_ were also found: *A. sisalana* (2.06%), H11648 (1.02%) and H400f (0.32%). These values support the hypothesis of a shift from growth metabolism to cell maintenance metabolism in the soil microbiome of the hybrids. The higher C_mic_/C_org_ value indicates more carbon available for microbial growth, suggesting a higher OM quality in the *A. sisalana* soil [141]. These results indicate that plant age and planting method are determining factors in the composition of the microbiota of these soils and that, over time, the microbiota interacting with these plants becomes more resilient and stable [75,142, 143].

We also evaluated significant differences in MBN (Fig. 3c) between *A. sisalana* (0.6 µmol N.g soil^−1^) and the hybrids H16648 (0.27 µmol N.g soil^−1^) and H400f (0.25 µmol N.g soil^−1^). These differences in MBN suggest a shift in the nitrogen use towards cellular maintenance in the hybrids, through protein accumulation or turnover and towards microbial growth in *A. sisalana* [32,144]. Furthermore, this finding supports the theory that microbial stress decreases alongside plant cultivation age [142,145].

## Vector analysis of ecoenzymes

To classify enzyme activities of carbon enzymes (CE), nitrogen enzymes (NE) and phosphorus enzymes (PE), we added the individual enzyme activity values for enzymes that supply the reference element and subtracted the enzyme activity values for enzymes that consume the element. Thus, CE=*β*-glucosidase+cellulase+xylanase−dehydrogenase; NE=urease+protease; and PE=acid phosphatase (Table 2).

We included both *β*-glucosidase and cellulase activities separately, despite *β*-glucosidase being part of the cellulase enzyme family, because of the distinct assay methods: *β*-glucosidase activity is maximized with P-nitrophenyl-*β*-d-glucose as substrate, while cellulase activity is detected through the 3,5-dinitrosalicylic acid method, which depends on cellobiose release for the glucose evolution through *β*-glucosidase activity. In soils, the synergistic activity of these carbon enzymes ensures maximum glucose release, due to the concentration of substrate for enzymatic action being divided into cellobiose-rich and cellobiose-poor microenvironments [146,148]. Urease is the primary enzyme releasing nitrogen in soils, followed by protease and chitinase [32]. The combination of urease and protease captures most of the nitrogen availability. And acid phosphatase was used to represent phosphorus availability due to the proximity of soil pH to the assay buffer (pH 6.5) (Table 1) used for phosphomonoesterase activity [117].

The values obtained for CE averaged 1.095 µmol product.g soil^−1^.h^−1^ for soils of the three *Agave* species evaluated, a value very close (≈1 : 1) to that typically found in native caatinga soils (0.65–1.2 µmol product.g soil^−1^.h^−1^) [149,150]. For NE, an average of 9.170 µmol product.g soil^−1^.h^−1^ was observed, slightly higher (+27.36 %) than the typical range for caatinga soils (3.88–7.2 µmol product.g soil^−1^.h^−1^) [149,151]. The average value of PE was 32.196 µmol product.g soil^−1^.h^−1^, representing a substantial increase of 129.97% compared to the typical range of values observed in native soils of the region (10.0–14.0 µmol product.g soil^−1^.h^−1^) [149,152].

Vector analysis of enzyme activity was performed to construct a graph in which the *y*-axis represents the percentage of carbon enzymes relative to carbon plus nitrogen enzymes (CE/(CE+NE)), and the *x*-axis represents the percentage of carbon enzymes relative to carbon plus phosphorus enzymes (CE/(CE+PE)) [34]. In this graph, vectors were drawn from the origin to the sample points, where vector length (VL) represents the strength of nutrient limitation and vector angle (VA) indicates the limitation by phosphorus (>45°), nitrogen (<45°) or enzymatic stoichiometry in a 1 : 1 : 1 ratio between carbon, phosphorus and nitrogen enzymes [34,153].

There were no significant differences in VL and VA among the soils of the three *Agave* species analysed (Table 2). The overall mean VL was 0.118, considered low, which suggests a slight carbon limitation [34]. The overall mean VA was 72.12°, greater than 45°, indicating phosphorus limitation in soils; although, due to the short vector length, this limitation is considered weak [124]. The vector data suggest that soils are not in 1 : 1 : 1 enzyme stoichiometry for carbon, phosphorus and nitrogen enzymes, with activity values for phosphorus enzymes being higher than those for nitrogen and both higher than those for carbon [34,154]. Furthermore, the numerically higher VL and VA values for the microbiota of the hybrids compared to those of *A. sisalana* indicate that the microbiota of *A. sisalana* may be more stable than that of the hybrids, but the lack of statistically significant difference does not allow confirmation of this result [155,156].

In comparison with other studies of soil enzymatic vectors in arid regions, we found VL values greater than 0.5 in areas with agricultural systems implemented in China, as well as VA values greater than 60°, which also indicates phosphorus limitation in the soil. However, this limitation is more severe than that observed in *Agave* plantations due to the higher VL values [103]. For natural arid climate areas with stony soils, severe nitrogen limitation was observed, with VL values greater than 1.1 and VA less than 40° [157]. In another natural area of desert steppe, the stoichiometry found deviates from the expected 1 : 1 : 1 ratio for C/N/P enzymes with the observed value being 1.2 : 1 : 1.5 [158]. These findings suggest that in arid regions, there is a climatic imbalance that causes shifts in the global pattern of enzyme stoichiometry, leading to either N or P limitations depending on the type of soil evaluated. Furthermore, in soils under agricultural use, there is a predominance of P limitations [103,157,159].

The restriction of phosphorus availability for micro-organisms in soil can result in metabolic deviations related to carbon and nitrogen cycles [46,103, 160]. As demonstrated in experiments involving the application of nitrogen fertilizers, the incorporation of N into soil leads to a relative increase in carbon mineralization, owing to the acceleration of microbial metabolism. Concurrently, this process results in P limitation due to the reduced mineralization rate of this nutrient [103]. This phenomenon can be observed through the analysis of the ratio between nitrogen and phosphorus enzymes (average NE/PE=0.304), with phosphatases exhibiting increased expression due to the necessity of acquiring more phosphorus to maintain C/N/P stoichiometry and microbial homeostasis [45]. The elemental balance of C, N and P in the soil has been shown to induce microbial activity in the production of enzymes for the acquisition of limiting nutrients. However, these nutrients may not be limiting for plant species, but rather for the microbial biomass present in the soil [46,160].

The *Agave* fields evaluated in this study did not receive nitrogen fertilizers; thus, the slight phosphorus limitation observed by the enzyme vectors is attributed to a cascade of nutritional accumulation by microbial biomass in the soil [45,46, 159,161]. The increased availability of a single nutrient (e.g. carbon, nitrogen or phosphorus) in the soil has been shown to stimulate the expression of nutrient acquisition enzymes, thereby enhancing the uptake of limiting nutrients [45,46, 160]. In this study, we theorized that initially elevated carbon levels in the soil (TOC>20 g.kg^−1^) promoted an increased expression of enzymes associated with biological nitrogen fixation, nitrification and denitrification [45,46, 160]. These enzymes function as a form of nitrogen fertilization in the soil, thereby increasing the available total nitrogen (TN>1,900 mg.kg^−1^). Consequently, phosphorus becomes the final limiting macronutrient for microbial acquisition, potentially resulting in an abundance of P-mineralizing and P-solubilizing micro-organisms within the microbiota of these soils, leading to elevated levels of phosphatase expression [103,160, 161]. This phenomenon has been observed in other natural or agronomic regions with arid climates, where phosphorus is also the limiting nutrient [45,103, 152, 157,159].

## Integrated microbiological soil analysis

In order to investigate the influence of chemical and biological parameters on the formation of the soils analysed, we carried out PCA of 25 variables. The integrated variables were CE, NE and PE (Table 2) [32,36, 112, 117, 162]. The ratios between carbon, nitrogen and phosphorus enzymes, e.g. CE/NE, CE/PE and NE/PE (Table 2) [32]. The VL and VA, calculated using the proportions between carbon and nitrogen (CE/(CE+NE)) and carbon and phosphorus (CE/(CE+PE)) enzymes (Table 2) [34]. The activity of alkaline phosphatases (AF) and arylsulphatases (SE) was used separately (Table 2) [128,163]. Soil chemical parameters are as follows: TOC, TN, phosphorus in soil (PS), calcium in soil (CaS), potassium in soil (KS), magnesium in soil (MgS), sodium in soil (NaS), CEC and pH in water (pH) (Table 1). And the ratios between carbon, nitrogen and phosphorus in soil, such as TOC/TN, TOC/PS and TN/PS, were used to compare the elemental ratios in soil with the enzymatic ratios [32,164, 165].

PCA fitted the variables with 50.22% of the variance explained by principal component (PC) 1 and 20.05% for PC2 (Table S1). The Shapiro–Wilk test indicated normality for PC2 (*P*-value=3.6e−1) and negative normality for PC1 (*P*-value=1.6e−2). Due to the non-parametrization of PC1, Levene’s test was used to assess the homogeneity of variances between samples (*P*-value PC1=8.9e−2 and *P*-value PC2=6.0e−2); after confirming the homogeneity of variances, we could proceed with the ANOVA for the principal components, even though PC1 was not parametric [166]. The ANOVA showed a significant difference between samples for PC1 (*P*-value=7.835e−7) and PC2 (*P*-value=2.62e−4) (Table S1), so we proceeded with Tukey’s test for multiple comparisons between *Agave* species (Table 3).

**Table 3.** Significance values for the Tukey test comparing *A. sisalana* soil, H11648 and H400f between PC1 and PC2 of the PCA

Comparison	***P*-value**	
	PC1	PC2
***A. sisalana*–H11648**	6.6e−6^***^	4.16e−2^**^
***A. sisalana*–H400f**	1.2e−6^***^	2.02e−2^**^
**H11648–H400f**	3.23e−01	1.84e−4^***^

* The asterisks represent the levels of statistical significance: **p ≤ 0.01, and ***p ≤ 0.001.

**Fig. 4.**
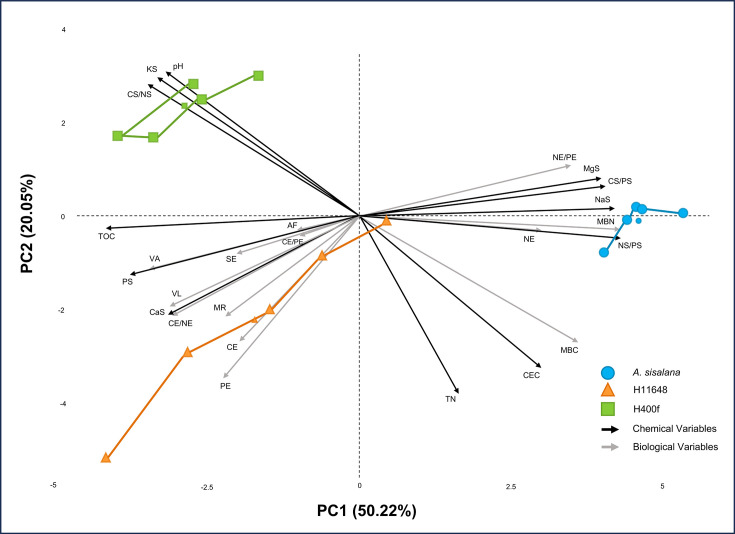
PCA for *A. sisalana* soil, H11648 and H400f. pH, pH in water; KS, soil potassium; CS/NS, TOC/TN; TOC, total organic carbon; AF, alkaline phosphatase; VA, vector angle; PS, phosphorus in soil; SE, arylsulphatase activity; CE/PE, carbon enzymes/phosphorus enzymes; VL, vector length; CaS, calcium in soil; CE/NE, carbon enzymes/nitrogen enzymes; MR, basal microbial respiration; CE, carbon enzymes; PE, phosphorus enzymes; TN, total nitrogen; CEC, cation exchange capacity; MBC, microbial biomass carbon; NS/PS, TN/PS; NE, nitrogen enzymes; MBN, microbial biomass nitrogen; NaS, sodium in soil; CS/PS, TOC/PS; MgS, magnesium in soil; NE/PE, nitrogen enzymes/phosphorus enzymes. The connection between the points on the graph indicates colour clustering (*k*=3).

For PC1, Tukey’s test significantly differentiated the *A. sisalana* soil from both hybrids, with no significant difference between the hybrids (Table 3), while for PC2, all three *Agave* species differed significantly (Table 3). These results can be interpreted to indicate that, for the majority of the variables (PC1=50.22%), the soil of the hybrids is indistinguishable from each other, and both of them are different from the *A. sisalana* soil. For the residual variance attributed to PC2 (20.05%), however, there is a clear distinction among all three soils (Fig. 4). This phenomenon can be explained by the establishment of the hybrid fields, where H11648 and H400f plants were cultivated in alternating rows of the same planting age, resulting in very similar soil conditions between hybrids [167,168]. In contrast, the *A. sisalana* field is considerably older than the hybrid fields and was planted with a randomly spaced pit disposition (not in rows), suggesting that the observed differences are not solely attributable to the plant itself but also to the time of cultivation and the management practices employed in the *Agave* fields [168,170].

A comparison between the ten variables that exerted the most significant influence on PC1 (in descending order: TN/PS, MBN, NaS, TOC, TOC/PS, MgS, PS, MBC, NE/PE and TOC/TN) and the ten variables that exhibited the most significant impact on PC2 (in descending order: TN, PE, CEC, pH, KS, TOC/TN, MBC, CE, MR and CE/NE) was conducted. The findings revealed that merely 30% of the variables that contributed most significantly to PC1 were of biological origin, with the remainder originating from chemical origin. In PC2, 50% of the variables that exerted the greatest influence were found to be biological. This observation suggests that the factors uniting the hybrid’s soils and distinguishing them from the *A. sisalana* soil in PC1 are predominantly of chemical origin, which may be attributable to variations in the geographical location of the fields and the distinct management practices of the plantations [167]. In the case of PC2, however, the soils of the three *Agave* species differ significantly, suggesting that biological factors play a more significant role in shaping this PC. Therefore, it can be inferred that the different *Agave* genotypes exert an influence on the microbiota of this soil, even in close proximity, such as the inter-rows where the hybrids are planted. Furthermore, with the passage of time, this biological influence of the plants on the soil becomes more evident, particularly when considering the differences in planting time between the hybrids and *A. sisalana* fields [168].

The absence of a discernible correlation between PC1 and PC2 (*P*-value=−2.45e−16) substantiates the hypothesis that the PCs are engaged in the processing of disparate data (Table S1). This finding serves to validate our hypothesis regarding the distinction between chemical and biological data. Moreover, the grouping of the data with *k*=3 results in the separation of the samples into three distinct groups of five samples each, thereby accurately distinguishing the three *Agave* species analysed (Table S1). These statistical confirmations lend robustness to our conclusions that different *Agave* genotypes exert an influence on the soil, with the exact separation of statistical groups between the *Agave* species analysed [170,171].

The RDA model yielded a *P*-value of 0.008 for the global ANOVA, indicating that TOC and TN are environmental variables that account for a substantial portion of the variance in the microbial community described by the model (Table S1). For the axes, RDA1 has a *P*-value of 0.02 (eigenvalue=79%), which is significant, and RDA2 has a *P*-value equal to 0.288 (eigenvalue=21%), which is not significant (Table S1). The TOC exhibited a substantial impact on the explained variance (*P*-value=0.008), while the TN demonstrated a non-significant influence (*P*-value=0.188) (Table S1). The partitioning of variance reveals that 32.98% of the observed variance is explained by TOC and TN, with the remainder (67.02%) unexplained by the environmental variables analysed (Table S1).

According to the literature, a value of 32.98% is considered a high proportion of the explained variance, given the soil’s inherent microenvironments that are in constant change, as it is virtually impossible to explain all the variance found in microbial communities in natural environments, such as soil, by measuring environmental variables in the laboratory [172,173]. The VIF is equal to 1.02 for TOC and TN, which is very close to 1, thereby strengthening the reliability of the model created, as TOC and TN are not collinear variables. The adjusted *R*^2^ was found to be 0.218, indicating that ~22% of the total variation in the response variables is actually attributable to TOC and TN. Consequently, the variation explained by RDA1 is actually 26%, while RDA2 accounts for 7% (Table S1).

Given the significance of RDA1 and TOC, it is concluded that TOC exerts a primary influence on the RDA1 axis, with the microbial community in the evaluated soils being strongly influenced by the available organic carbon content. This finding aligns with the conclusions of earlier ecological studies, which demonstrated that an elevated carbon content in the soil leads to the expression of enzymes responsible for acquiring additional elements to maintain the C/N/P balance within the environment and the intracellular space of soil micro-organisms [45,46]. The high carbon content in the environment functions as a driver for the availability and acquisition of other elements, including N and P [46]. Also, according to the RDA model, TN represents a smaller fraction of the explained variance, yet it still influences the evaluated response variables in some manner, being the second most important nutrient in the evaluated soils [174,175].

PERMANOVA was performed using the Euclidean distance metric with separation of the three groups evaluated (soil from *A. sisalana*, H11648 and H400f), using all 25 variables included in the PCA for statistical calculation. The calculated *R*^2^ was equal to 0.6515 (Table S1), with 65% of the variation in the data explained by the differentiation between *Agave* species. This highlights the genotypic factor of these plants in fostering distinct relationships with the microbiota with which they interact [16,17, 19, 23, 70]. The *P*-value for the *F* test was 0.001 (Table S1), indicating a highly significant result, confirming the hypothesis that the three *Agave* species exhibit significant differences according to the Euclidean matrix generated. To validate the test, a dispersion test was performed, resulting in a *P*-value of 0.4502 (Table 1). This indicates that the dispersions within the groups do not differ statistically, thereby corroborating the result found by PERMANOVA.

Since 65% of the variation in the data was explained solely by the grouping and differentiation between the analysed *Agave* species, we infer that the remaining variation may be due to the different planting methods used for the *Agave* hybrids compared to *A. sisalana*, as well as the longer cultivation time of *A. sisalana* compared to the hybrids. As observed by RDA, the environmental variable TOC significantly impacts the variation in response data. Therefore, we can conclude that as *A. sisalana* soils are older and have less available organic carbon, the active microbiota shifts the C/N/P balance closer to 1 : 1 : 1 over time by capturing limiting nutrients (first N and then P) while consuming the excess C. Further studies of planting areas older than *A. sisalana* are needed to determine if this balance is maintained over time or if it masks a slow depletion of nutrients in the soil.

It can be concluded that the three *Agave* species exhibit distinct patterns of soil shaping and microbiota interaction, either in confined geographical areas, as observed in the case of the hybrids field, or in response to varying management and planting schemes, as evidenced by the *A. sisalana* field [170,176]. According to the most recent scholarly literature on the subject, the studied *Agave* plantations have been found to maintain soil in a fertile state for the cultivation of most plant species of agricultural interest [177,178], even in the absence of agronomic management to replenish soil nutrients and with intensive biomass removal, such as biannual leaf cutting for the production of natural fibres in these plantations. However, further studies in other regions with *Agave* plantations are necessary to rule out the hypothesis that eutrophic soils solely maintain the nutritional balance of these plantations. Additionally, studies of even longer cultivated *Agave* plantations are required to confirm that the assessed elemental balance is maintained over many years of agronomic exploitation.

## Microbial quantification and soil parameter correlation

**Fig. 5.**
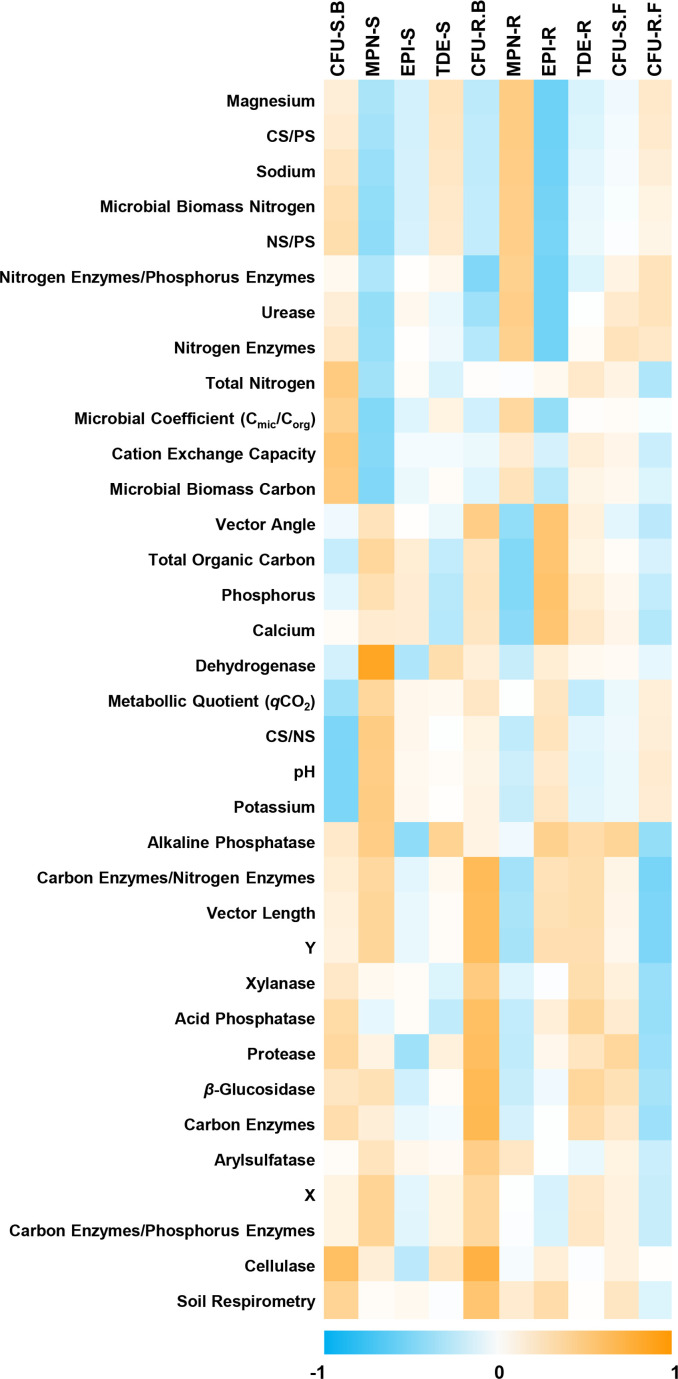
Correlation between four methods for quantification of soil and rhizosphere micro-organisms of *Agave* spp*.* (CFU.S.B, soil bacterial colony-forming unit counting; MPN.S, soil most probable number; EPI.S, soil epifluorescence microscopy; TDE.S, soil total DNA extraction; CFU.R.B, rhizosphere bacterial colony-forming unit counting; MPN. R, rhizosphere most probable number; EPI.R, rhizosphere epifluorescence microscopy; TDE.R, rhizosphere total DNA extraction; CFU.S.F, soil fungal colony-forming unit counting; CFU.R.F, rhizosphere fungal colony-forming unit counting; CS/PS, total organic carbon/phosphorus in soil; CS/NS, total organic carbon/total nitrogen; NS/PS, total nitrogen/phosphorus in soil). Variables were grouped by calculating the Euclidean distance of the correlation matrix.

Given the absence of statistically significant variations in the quantification of micro-organisms in the soil and the *Agave* rhizosphere for the three *Agave* species examined, including these variables in the PCA would have been redundant. Therefore, to integrate the microbial quantification data with the biological and chemical data analysed for the soil, we performed correlation analyses between these variables (Fig. 5).

A significant number of studies have sought to establish a correlation between quantitative data on soil microbiota and analyses of enzymatic activities and biological parameters in soil, yet the results have shown substantial variability [41,43,179]. This complicates the determination of the role of micro-organism numbers in shaping the surrounding environment. However, a limited population of micro-organisms is insufficient to effectively support energy and nutrient cycling in the soil environment [180].

Some studies have observed a negative correlation with urease activity and microbial quantification [43,179], and in our case, the only significant correlation (*P*-value=4.60e−2) for urease was negative (Spearman=−0.521) (Table 4), specifically with bacterial quantification in the rhizosphere by EPI. Other studies have found a strong correlation between dehydrogenase activity and microbial quantification [41,179], and in our study, the only significant correlation for dehydrogenase was with the bacterial quantification in the rhizosphere by c.f.u. counting (Spearman=0.568) (Table 4). Significant correlations have also been observed between phosphatase activity and microbial quantification [41,42], but in our analysis, no significant association was detected.

**Table 4.** Spearman correlations between different soil microbial quantification methods and soil chemical and biological parameters

Variable 1 – variable 2	Spearman correlation	***P*-value**
**Cellulase – c.f.u. (soil – bacteria)**	0.604	0.017^*^
**Total organic carbon in soil – EPI (rhizosphere)**	0.586	0.022^*^
**Phosphorus in soil – EPI (rhizosphere)**	0.586	0.022^*^
**Calcium in soil – EPI (rhizosphere)**	0.586	0.022^*^
**Magnesium in soil – EPI (rhizosphere)**	−0.586	0.022^*^
**Sodium in soil – EPI (rhizosphere)**	−0.586	0.022^*^
**CS/PS – EPI (rhizosphere)**	−0.586	0.022^*^
**Dehydrogenase – c.f.u. (rhizosphere – bacteria)**	0.568	0.027^*^
**Cellulase – c.f.u. (rhizosphere – bacteria)**	0.546	0.035^*^
**Protease – EPI (soil)**	−0.525	0.045^*^
**Vector angle – c.f.u. (rhizosphere – bacteria)**	0.525	0.045^*^
**Urease – EPI (rhizosphere)**	−0.521	0.046^*^
**NE/PE – EPI (rhizosphere)**	−0.521	0.046^*^

* Significant correlation at *p* < 0.05.

CFU, colony-forming unit counting; CS/PS, carbon in soil/phosphorus in soil; EPI, epifluorescence microscopy; NE/PE, nitrogen enzymes/phosphorus enzymes.

Of the 13 correlations with significant values (both negative and positive), 8 are of variables correlated with the quantification by EPI of prokaryotes in the *Agave* rhizosphere (Table 4). These included TOC, PS, CaS, MgS, NaS, urease, the nitrogen-to-phosphorus enzyme ratio and the soil carbon-to-phosphorus ratio. This indicates that this quantification method, specifically for the rhizosphere, can be used to estimate biological activity and chemical conditions in the analysed soils, especially due to the positive correlation between EPI and TOC, since we assessed that this environmental variable is responsible for most of the variability observed in our response variables. This finding may be related to the greater availability of nutrients in the rhizosphere from plant exudates, which are converted into microbial enzymatic activity, affecting the chemical parameters of the soil and highlighting the role of the plant in shaping its microbiota and the fertility of the environment in which it lives [181].

## Supplementary material

10.1099/mic.0.001681 Uncited Supplementary Material 1.

## Abbreviations

CAM: crassulacean acid metabolism
CaS: calcium in soil
CE: carbon enzymes
CEC: cation exchange capacity
DRBC: dichloran rose bengal chloramphenicol
EEAs: ecoenzymes
EPI: epifluorescence microscopy
GHG: greenhouse gas
INT: iodonitrotetrazolium chloride
KS: potassium in soil
m%: aluminium saturation
MBC: microbial biomass carbon
MBN: microbial biomass nitrogen
MgS: magnesium in soil
MPN: most probable number
MR: microbial respiration
NaS: sodium in soil
NE: nitrogen enzymes
OM: organic matter
P: phosphorus
PC: principal component
PCA: principal component analysis
PE: phosphorus enzymes
pH: pH in water
PS: phosphorus in soil
SB: sum of bases
TDE: total DNA extraction
TN: total nitrogen
TOC: total organic carbon
TSA: tryptic soy agar
TTF: triphenyltetrazolium formazan
V%: base saturation
VA: vector angle
VIF: variance inflation factor
VL: vector length
