# Supplementary material for: An integrated microbiological analysis of the soil and rhizosphere of Agave spp. under minimum technological input farming systems
Source: Microbiology (Reading). 2026 May 15;172(5):001681. doi: 10.1099/mic.0.001681 (PMC13178802; doi:10.1099/mic.0.001681)

Supplementary 1 (Table 1): Values for enzyme activity, enzyme stoichiometry calculations and soil microbiological parameters analyzed for five biological soil replicates of *Agave sisalana*, *Agave* Hybrid H11648 and *Agave* Hybrid H400f. SD = Standard Deviation.

Soil Enzymatic Activity

Agave Sample	β-Glucosidase (BG)	Arylsulfatase (SE)	Alkaline Phosphatase (AlkP)	Acid Phosphatase (AcdP)	Protease (PO)	Urease (UR)	Cellulase (CL)	Xylanase (XY)	Dehydrogenase (DH)
	μmol of p-Nitrophenol/(g of soil.h)				μmol of L-Tyrosine/(g of soil.h)	μmol of NH_4^+-N/(g of soil.h)	μmol of Glucose/(g of soil.h)		μmol of TTF/(g of soil.h)
***A. sisalana* - 1**	0.247	1.137	30.418	28.206	0.163	8.791	0.164	0.327	0.002
***A. sisalana* - 2**	0.315	1.707	30.931	23.290	0.009	9.667	0.094	0.447	0.001
***A. sisalana* - 3**	0.365	2.752	36.783	29.910	0.238	11.042	0.237	0.565	0.002
***A. sisalana* - 4**	0.456	2.264	58.140	26.442	0.105	15.376	0.052	0.493	0.004
***A. sisalana* - 5**	0.408	0.960	46.954	29.227	0.570	9.000	0.183	0.125	0.002
**Mean**	0.358	1.764	40.645	27.415	0.217	10.775	0.146	0.391	0.002
**SD**	0.081	0.753	11.829	2.650	0.215	2.718	0.073	0.172	0.001
**H11648 - 1**	0.307	1.526	32.524	28.135	0.667	11.694	0.083	0.242	0.005
**H11648 - 2**	0.352	1.757	45.884	35.926	0.484	9.540	0.182	0.776	0.001
**H11648 - 3**	0.343	0.797	35.276	31.695	0.402	8.491	0.212	0.068	0.003
**H11648 - 4**	0.962	3.282	45.836	58.124	1.723	6.508	0.329	1.537	0.003
**H11648 - 5**	0.493	2.316	48.332	45.445	0.090	5.850	0.053	0.745	0.001
**Mean**	0.491	1.936	41.570	39.865	0.673	8.417	0.172	0.673	0.002
**SD**	0.273	0.929	7.141	12.087	0.623	2.358	0.110	0.573	0.002
**H400f - 1**	0.584	0.975	41.170	29.001	0.132	11.215	0.027	0.811	0.005
**H400f - 2**	0.593	2.382	38.577	30.066	0.157	7.873	0.117	0.195	0.008
**H400f - 3**	0.627	3.072	32.198	36.402	0.442	6.772	0.026	0.532	0.002
**H400f - 4**	0.572	3.397	68.388	24.824	0.355	4.517	0.166	0.374	0.018
**H400f - 5**	0.372	1.942	41.691	26.244	0.097	5.578	0.095	0.227	0.002
**Mean**	0.550	2.354	44.405	29.308	0.237	7.191	0.086	0.428	0.007
**SD**	0.101	0.958	13.928	4.486	0.153	2.578	0.060	0.253	0.006
**General Mean**	0.466	2.018	42.207	32.196	0.376	8.794	0.135	0.497	0.004
**General SD**	0.181	0.858	10.617	9.036	0.422	2.823	0.086	0.371	0.004

Soil Enzymatic Calculations

Agave Sample	Carbon Enzymes (CE)	Phosphorus Enzymes (PE)	Nitrogen Enzymes (NE)	Enzymes Ratios			Enzymes Proportions		Vector Lenght (VL)	Vector Angle (VA)
	CE = (BG + CL + XY - DH)	PE = AcdP	NE = (UR + PO)	CE/PE	CE/NE	NE/PE	X = CE/(CE + PE)	Y = CE/(CE + NE)	VL = $(X^2 + Y^2)^{1/2}$	VA = Degrees(ATAN2(X;Y))
***A. sisalana* - 1**	0.735	28.206	8.954	0.026	0.082	0.317	0.025	0.076	0.080	71.489
***A. sisalana* - 2**	0.855	23.290	9.676	0.037	0.088	0.415	0.035	0.081	0.089	66.436
***A. sisalana* - 3**	1.165	29.910	11.280	0.039	0.103	0.377	0.037	0.094	0.101	68.175
***A. sisalana* - 4**	0.997	26.442	15.481	0.038	0.064	0.585	0.036	0.060	0.071	59.014
***A. sisalana* - 5**	0.714	29.227	9.571	0.024	0.075	0.327	0.024	0.069	0.073	71.043
**Mean**	0.893	27.415	10.992	0.033	0.083	0.405	0.032	0.076	0.083	67.231
**SD**	0.189	2.650	2.652	0.007	0.015	0.109	0.007	0.012	0.012	5.042
**H11648 - 1**	0.626	28.135	12.362	0.022	0.051	0.439	0.022	0.048	0.053	65.698
**H11648 - 2**	1.308	35.926	10.024	0.036	0.130	0.279	0.035	0.115	0.121	73.072
**H11648 - 3**	0.620	31.695	8.893	0.020	0.070	0.281	0.019	0.065	0.068	73.596
**H11648 - 4**	2.826	58.124	8.231	0.049	0.343	0.142	0.046	0.256	0.260	79.718
**H11648 - 5**	1.290	45.445	5.940	0.028	0.217	0.131	0.028	0.178	0.181	81.206
**Mean**	1.334	39.865	9.090	0.031	0.162	0.254	0.030	0.133	0.136	74.658
**SD**	0.900	12.087	2.360	0.012	0.120	0.126	0.011	0.085	0.085	6.173
**H400f - 1**	1.416	29.001	11.347	0.049	0.125	0.391	0.047	0.111	0.120	67.238
**H400f - 2**	0.897	30.066	8.029	0.030	0.112	0.267	0.029	0.101	0.105	73.918
**H400f - 3**	1.183	36.402	7.214	0.032	0.164	0.198	0.031	0.141	0.144	77.406
**H400f - 4**	1.094	24.824	4.872	0.044	0.225	0.196	0.042	0.183	0.188	77.036
**H400f - 5**	0.693	26.244	5.675	0.026	0.122	0.216	0.026	0.109	0.112	76.700
**Mean**	1.057	29.308	7.427	0.036	0.149	0.254	0.035	0.129	0.134	74.460
**SD**	0.276	4.486	2.518	0.010	0.046	0.082	0.009	0.034	0.034	4.266
**General Mean**	1.095	32.196	9.170	0.033	0.131	0.304	0.032	0.113	0.118	72.116
**General SD**	0.547	9.036	2.772	0.009	0.078	0.123	0.009	0.056	0.056	6.012

Soil Microbiological Parameters

Agave Sample	Soil Basal Microbial Respiration (MR)	Microbial Biomass Carbon (MBC)	Microbial Biomass Nitrogen (MBN)	Metabolic Quotient (qCO_2)	Microbial Coefficient (C_{mic}/C_{org})
	μmol of CO_2/(g of soil.day)	μmol of C/(g of soil)	μmol of N/(g of soil)	mg of CO_2/(g of MBC.h)	Percentage (%)
***A. sisalana* - 1**	0.785			3.214	
***A. sisalana* - 2**	1.298			5.309	
***A. sisalana* - 3**	1.830	37.311	0.636	7.486	2.1
***A. sisalana* - 4**	1.558			6.375	
***A. sisalana* - 5**	1.483			6.067	
**Mean**	1.391	-	-	5.690	-
**SD**	0.389	-	-	1.590	-
**H11648 - 1**	1.101			6.004	
**H11648 - 2**	2.143			11.682	
**H11648 - 3**	1.878	28.007	0.272	10.234	1
**H11648 - 4**	2.440			13.297	
**H11648 - 5**	1.826			9.952	
**Mean**	1.878	-	-	10.234	-
**SD**	0.498	-	-	2.714	-
**H400f - 1**	0.643			11.602	
**H400f - 2**	2.000			36.094	
**H400f - 3**	1.937	8.458	0.258	34.965	0.3
**H400f - 4**	1.896			34.218	
**H400f - 5**	1.625			29.328	
**Mean**	1.620	-	-	29.242	-
**SD**	0.565	-	-	10.194	-
**General Mean**	1.630	24.592	0.389	15.055	0.011
**General SD**	0.497	14.726	0.214	12.001	0.009

Supplementary 1 (Figure 1): Graphs for enzyme activity (µmol of product/(g of soil.h)) for the nine different soil enzymes evaluated for *Agave sisalana*, *Agave* H11648 and *Agave* H400f. The error bars represent the standard error of the sample set.

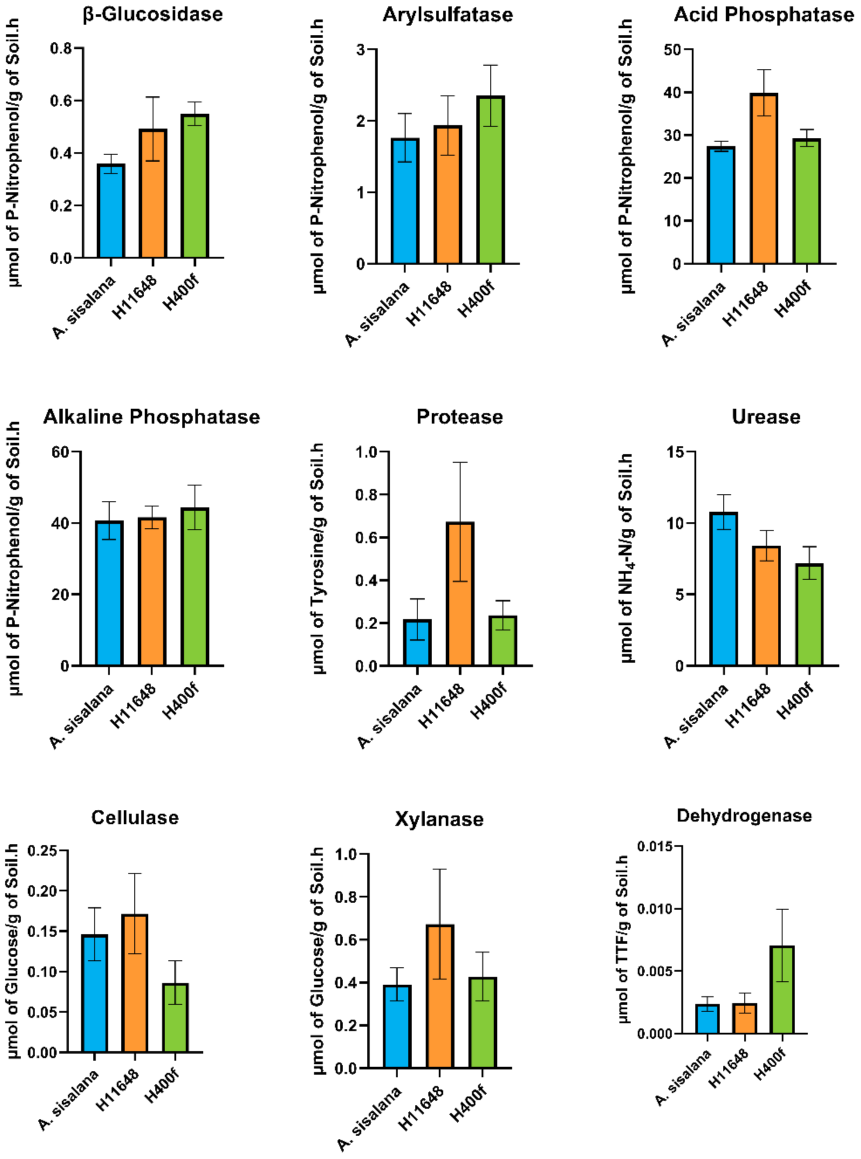

Supplementary 1 (Figure 2): Standard curves used to evaluate the enzymatic activity of the nine soil enzymes evaluated for *Agave sisalana*, *Agave* H11648 and *Agave* H400f.

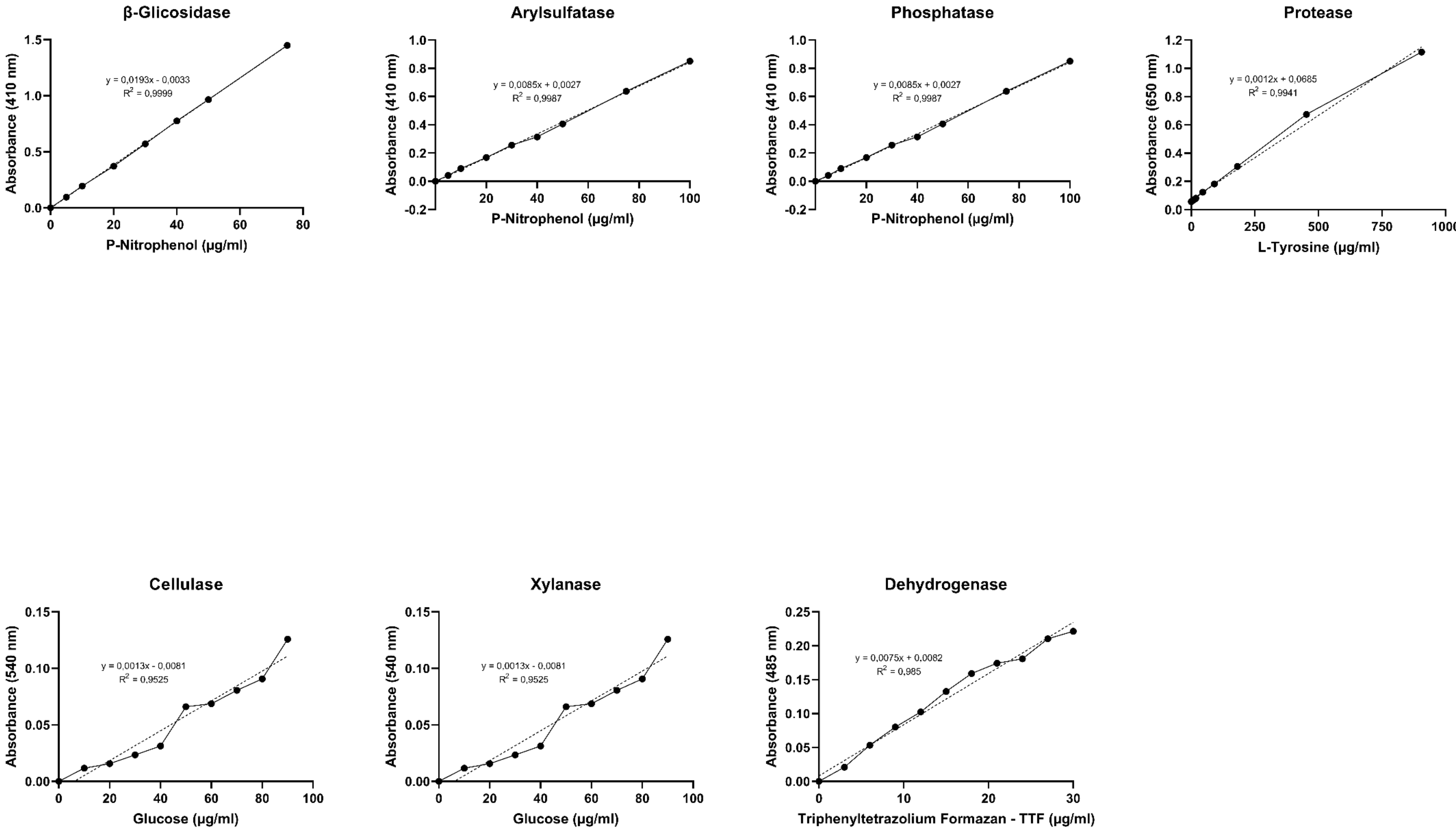

Supplementary 2 (Table 1): Results for quantification of soil and rhizosphere microorganisms of *Agave sisalana*, *Agave* H11648 and *Agave* H400f by five different microbiological quantification methods. EPI = Epifluorescence microscopy counting method; TDE = Total DNA extraction counting method; CFU-Bac = Colony counting method in culture medium for bacteria; CFU-Fungi = Colony counting method in culture medium for filamentous fungi and yeasts; MPN = Most probable number estimation method. SD = Standard Deviation.

***Agave* Sample**	**EPI**	**TDE**	**CFU-Bac**	**CFU-Fungi**	**MPN**
	log_{10}(CFU)/g of soil				log_{10}(MPN)/g of soil
***A. sisalana* - 1 (Soil)**	9,241	12,569	5,602	4,000	6,431
***A. sisalana* - 2 (Soil)**	9,405	12,484	6,778	4,505	6,431
***A. sisalana* - 3 (Soil)**	9,289	12,390	7,716	5,602	6,462
***A. sisalana* - 4 (Soil)**	9,146	12,712	7,549	5,602	5,462
***A. sisalana* - 5 (Soil)**	7,040	12,608	7,595	6,342	6,279
***A. sisalana* (Soil) - Mean**	**8,824**	**12,553**	**7,048**	**5,210**	**6,213**
***A. sisalana* (Soil) - SD**	**1,002**	**0,122**	**0,889**	**0,942**	**0,426**
**H11648 - 1 (Soil)**	8,814	12,332	5,510	5,603	6,886
**H11648 - 2 (Soil)**	9,103	12,330	5,985	5,173	6,653
**H11648 - 3 (Soil)**	9,498	12,665	8,466	5,011	6,857
**H11648 - 4 (Soil)**	8,595	12,596	8,330	5,570	7,000
**H11648 - 5 (Soil)**	9,447	12,193	6,704	5,208	6,462
**H11648 (Soil) - Mean**	**9,091**	**12,423**	**6,999**	**5,313**	**6,772**
**H11648 (Soil) - SD**	**0,392**	**0,199**	**1,347**	**0,261**	**0,214**
**H400f - 1 (Soil)**	8,784	12,420	5,991	5,371	7,886
**H400f - 2 (Soil)**	9,368	12,179	6,176	4,821	7,146
**H400f - 3 (Soil)**	9,342	12,455	5,471	5,231	6,079
**H400f - 4 (Soil)**	8,171	12,822	6,264	5,244	10,041
**H400f - 5 (Soil)**	9,285	12,701	5,707	5,138	6,079
**H400f (Soil) - Mean**	**8,990**	**12,515**	**5,922**	**5,161**	**7,446**
**H400f (Soil) - SD**	**0,516**	**0,252**	**0,330**	**0,207**	**1,640**
**Soil - Mean**	8,969	12,497	6,656	5,228	6,811
**Soil - SD**	0,648	0,192	1,032	0,538	1,051
***A. sisalana* - 1 (Rhizosphere)**	8,551	11,275	7,050	5,864	6,204
***A. sisalana* - 2 (Rhizosphere)**	8,370	11,222	6,290	5,130	6,672
***A. sisalana* - 3 (Rhizosphere)**	8,417	11,099	7,801	6,400	8,380
***A. sisalana* - 4 (Rhizosphere)**	8,279	11,642	6,573	5,487	7,924
***A. sisalana* - 5 (Rhizosphere)**	9,212	12,036	6,661	5,219	6,763
***A. sisalana* (Rhizosphere) - Mean**	**8,566**	**11,455**	**6,875**	**5,620**	**7,189**
***A. sisalana* (Rhizosphere) - SD**	**0,374**	**0,382**	**0,585**	**0,521**	**0,919**
**H11648 - 1 (Rhizosphere)**	8,982	11,208	6,325	5,433	6,462
**H11648 - 2 (Rhizosphere)**	10,132	11,328	6,617	5,635	7,279
**H11648 - 3 (Rhizosphere)**	9,184	11,855	7,674	5,696	5,362
**H11648 - 4 (Rhizosphere)**	8,922	11,811	8,921	4,882	5,886
**H11648 - 5 (Rhizosphere)**	9,701	12,104	6,814	4,813	6,000
**H11648 (Rhizosphere) - Mean**	**9,384**	**11,661**	**7,270**	**5,292**	**6,198**
**H11468 (Rhizosphere) - SD**	**0,519**	**0,378**	**1,051**	**0,418**	**0,720**
**H400f - 1 (Rhizosphere)**	8,695	12,070	6,945	5,684	5,544
**H400f - 2 (Rhizosphere)**	8,723	10,996	7,451	6,376	7,568
**H400f - 3 (Rhizosphere)**	8,412	11,688	6,972	5,423	6,792
**H400f - 4 (Rhizosphere)**	9,790	11,702	7,431	4,949	5,653
**H400f - 5 (Rhizosphere)**	9,928	10,599	6,934	6,051	6,792
**H400f (Rhizosphere) - Mean**	**9,110**	**11,411**	**7,146**	**5,697**	**6,470**
**H400f (Rhizosphere) - SD**	**0,697**	**0,597**	**0,269**	**0,553**	**0,857**
**Rhizosphere - Mean**	9,020	11,509	7,097	5,536	6,619
**Rhizosphere - SD**	0,616	0,444	0,680	0,498	0,887
**General Mean**	**8,994**	**12,003**	**6,877**	**5,382**	**6,715**
**General SD**	**0,622**	**0,605**	**0,888**	**0,533**	**0,961**

Supplementary 2 (Figure 1): Example of plates used to quantify bacteria (Tryptic Soy Agar culture medium) and quantify filamentous fungi and yeasts (Dichloran Rose Bengal Chloramphenicol culture medium) from the soil and rhizosphere of *Agave sisalana*, *Agave* H11648 and *Agave* H400f, using the colony count method (CFU).

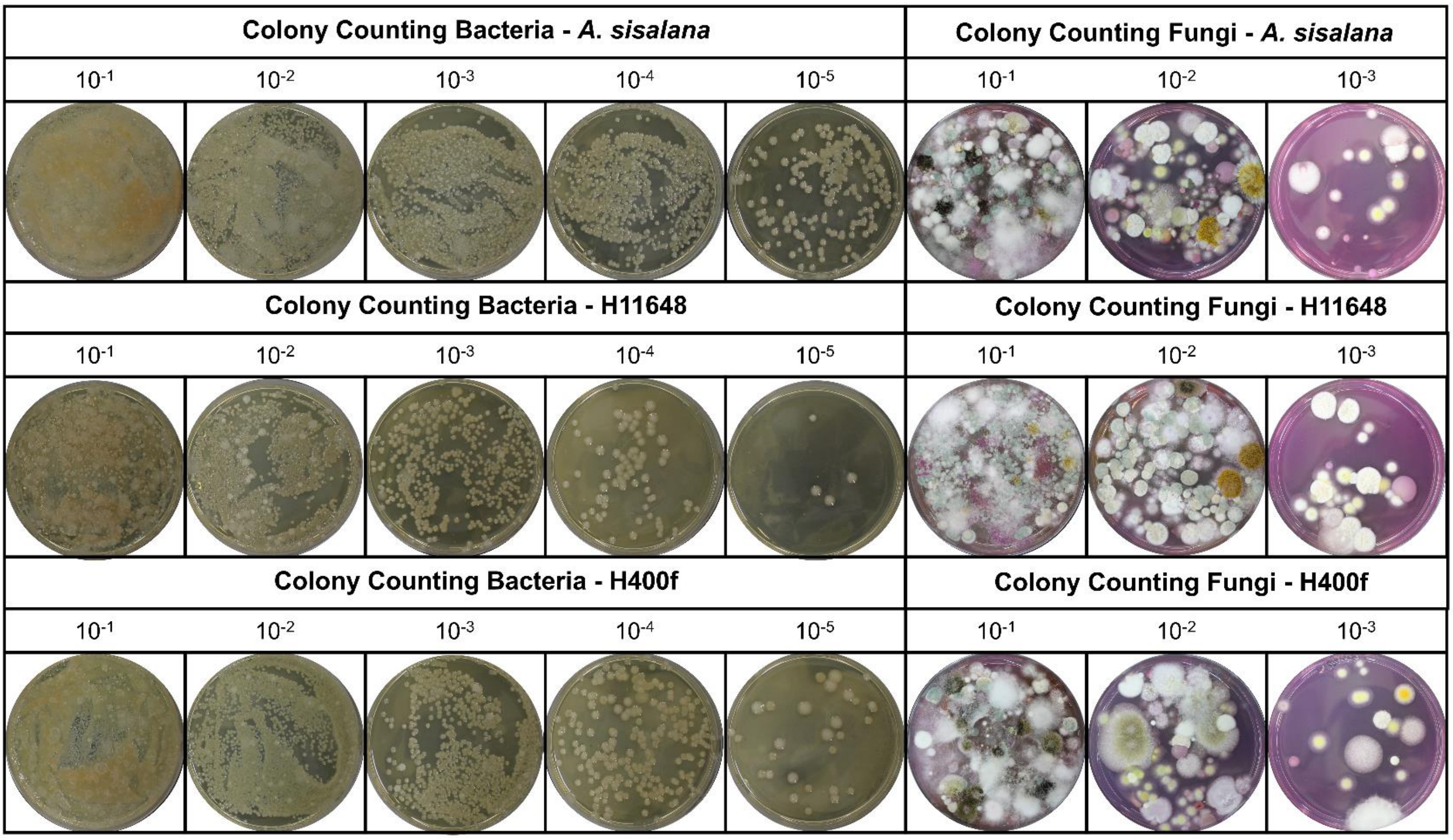

Supplementary 2 (Figure 2): Example of microplates used for the estimation of aerobic microorganisms in soil and rhizosphere of *Agave sisalana*, *Agave* H11648 and *Agave* H400f, using the most probable number (MPN) estimation method. Red wells indicate positive growth, due to a reduction in the Iodonitrotetrazolium Chloride (INT) reagent used as a growth-confirming agent. Tryptic Soy Broth was used as the culture medium.

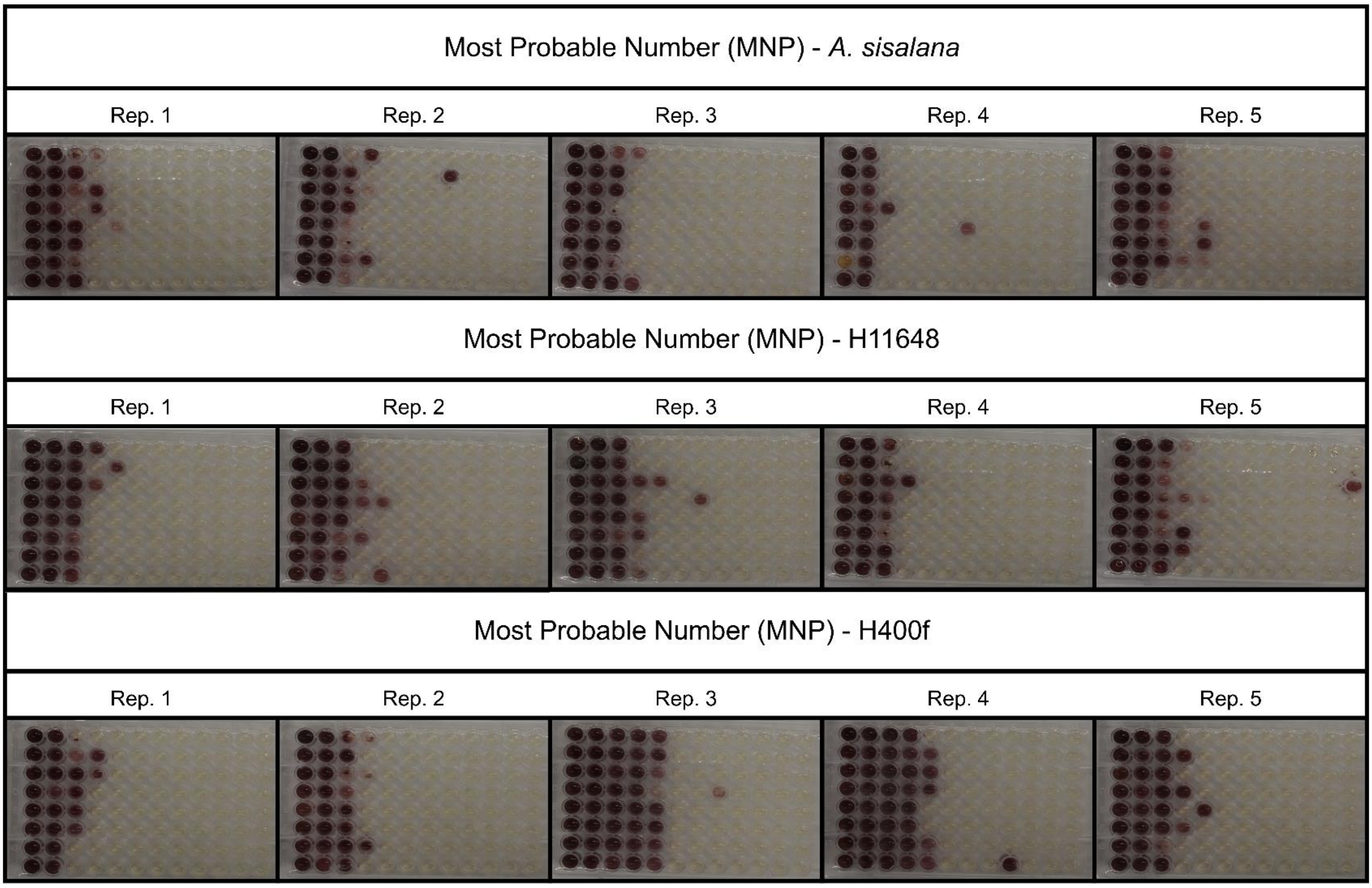

Supplementary 2 (Figure 3): Example of images generated by epifluorescence microscopy method of quantifying microorganisms in soil and rhizosphere of *Agave sisalana*, *Agave* H11648 and *Agave* H400f. Green fluorescence images are generated using the fluorophore Syto-9 and red fluorescence images are generated using the fluorophore Proidium Iodide (PI). Black and white images are generated by watershed image analysis software.

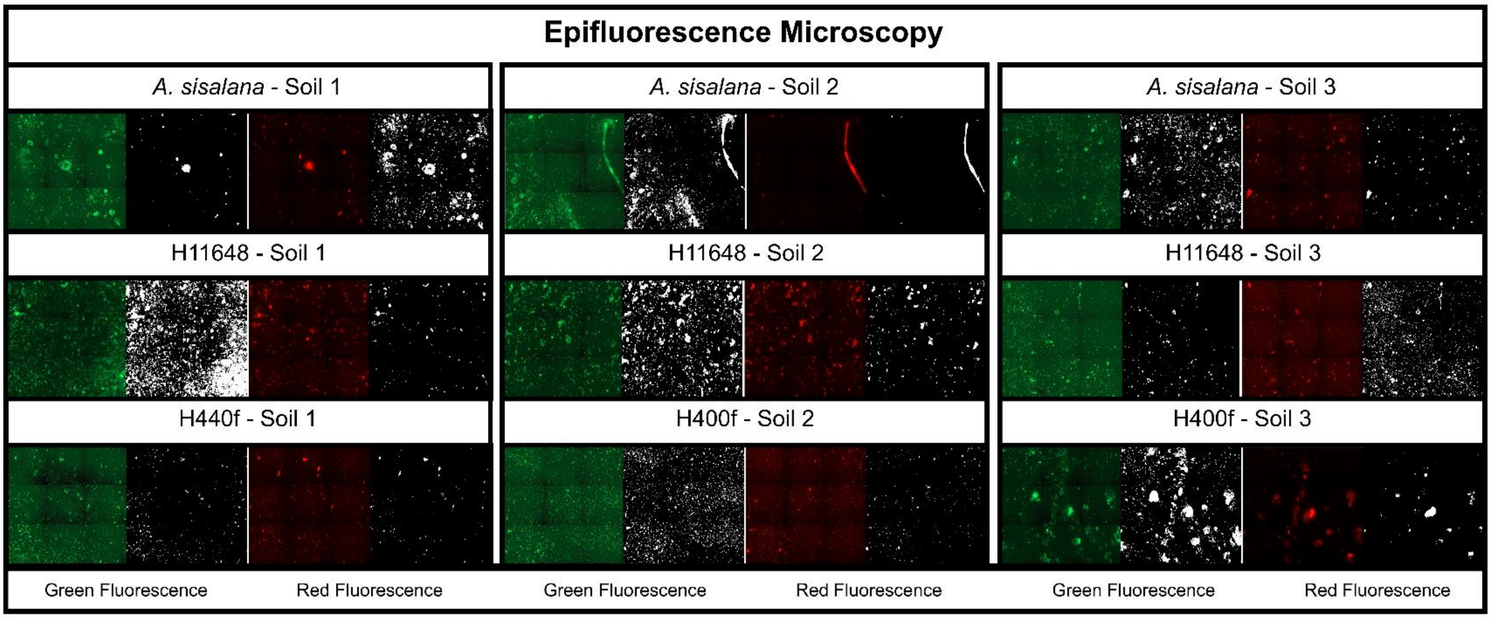

Supplementary 3 (Table 1): Statistical tests performed for the principal component analysis, redundancy analysis and PERMANOVA of the 25 chemical and biological soil variables of *Agave sisalana*, *Agave* H11648 and *Agave* H400f. PC = Principal Component; S = *A. sisalana* sample group; H = H11648 sample group; F = H400f sample group; TOC = Total Organic Carbon; TN = Total Nitrogen; VIF - Variance Inflation Factor; DF = Degrees of Freedom; SOS = Sum of Squares. * = 0.05 - 0.01; ** = 0.01 - 0.001; *** = < 0.001; ns = non-significative with 5% confidence.

***Agave* Sample**	**Principal Component Value**		
	PC1	PC2	PC3
***A. sisalana* - 1**	4,511	0,193	-0,003
***A. sisalana* - 2**	4,618	0,127	0,738
***A. sisalana* - 3**	3,997	-0,788	1,831
***A. sisalana* - 4**	5,292	0,050	1,184
***A. sisalana* - 5**	4,364	-0,101	0,310
**H11648 - 1**	0,441	-0,132	-3,998
**H11648 - 2**	-1,460	-2,026	-1,464
**H11648 - 3**	-0,612	-0,877	-3,645
**H11648 - 4**	-4,133	-5,203	2,342
**H11648 - 5**	-2,805	-2,943	-0,552
**H400f - 1**	-1,641	3,000	-0,051
**H400f - 2**	-2,573	2,486	-0,021
**H400f - 3**	-3,366	1,682	0,907
**H400f - 4**	-3,941	1,718	2,693
**H400f - 5**	-2,693	2,814	-0,270

**Percentage of Variance Explained**		**Shapiro-Wilk (p-value)**	**ANOVA (p-value)**	**Levene (p-value)**	**Kruskal-Wallis (p-value)**
PC1	50,22%	0,016^{ns}	7,835e-7***	0,089*	7,521e-3**
PC2	20,05%	0,356*	2,617e-4***	0,060*	2,454e-3**
PC3	14,50%	0,165*	0,101^{ns}	-	-
PC4	7,11%	-	-	-	-
PC5	3,84%	-	-	-	-

**Tukey p-value (PC1)**		**Dunn p-value (PC1)**		**Correlation**	
S-H	6,6e-6***	S-H	0,059^{ns}	PC1-PC2	-2,447e-16***
S-F	1,2e-6***	S-F	8,938e-3**	PC1-PC3	-9,351e-18***
H-F	0,323^{ns}	H-F	1^{ns}	PC2-PC3	-4,598e-17***

**Tukey p-value (PC2)**		**Dunn p-value (PC2)**		**Tukey p-value (PC3)**	
S-H	0,042*	S-H	0,312^{ns}	S-H	0,128^{ns}
S-F	0,020*	S-F	0,198^{ns}	S-F	0,988^{ns}
H-F	1,838e-4***	H-F	1,592e-3**	H-F	0,162^{ns}

Redundancy Analysis - RDA

**Test**	**Reference**	**p-value**	**Test**	**Value**
**ANOVA-Global**	Whole Model	0.008**	**Eigenvalue (RDA1)**	79%
**ANOVA_Axes**	RDA1	0.02*	**Eigenvalue (RDA2)**	21%
	RDA2	0.288^{ns}		
**ANOVA-Terms**	TOC	0.008**	**VIF - TOC**	1.02
	TN	0.188^{ns}	**VIF - TN**	1.02
**Partioning of Variance**	Total (13.0)	100%		
	Constrained (4.288)	32.98%	**R^2 - adjusted**	0.218
	Unconstrained (8.712)	67.02%		

PERMANOVA

-	**DF**	**SOS**	**R^2**	**F value**	**p-value (F)**
**Model**	2	228.03	0.652	11.217	0.001***
**Residual**	12	121.97	0.349		
**Total**	14	350.0	1		

Dispersion Test

**F value**	0.8535	**p-value (F)**	0.4502*

Supplementary 3 (Figure 1): Clustering with k = 3 for principal components (PC) 1 and 2 of the principal component analysis carried out with the 25 chemical and biological soil variables of *Agave sisalana*, *Agave* H11648 and *Agave* H400f.

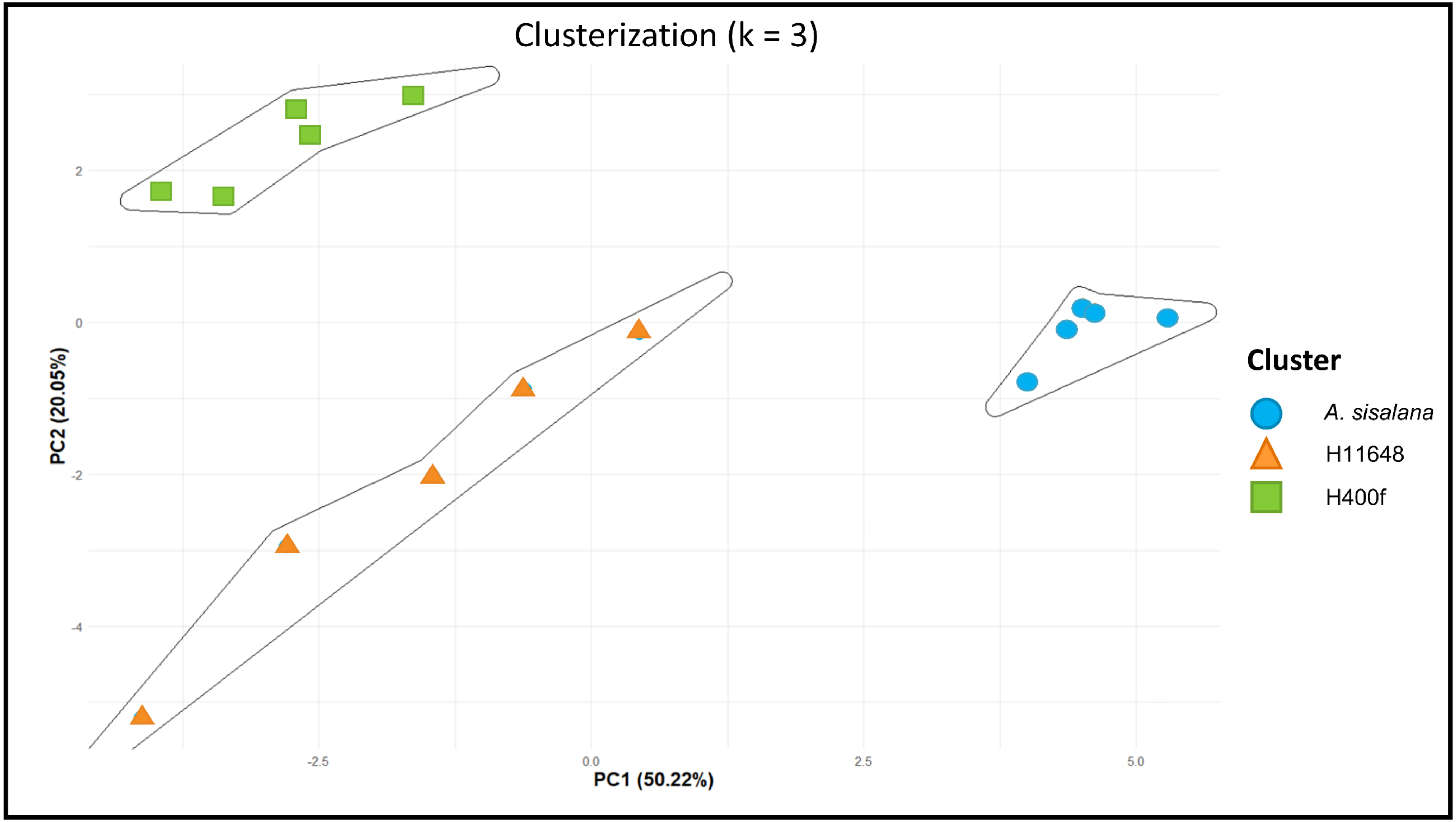

Supplementary 3 (Figure 2): Pearson's correlation between the 25 chemical and biological variables used for the principal component analysis of *Agave sisalana*, *Agave* H11648 and *Agave* H400f soil. pH = pH in water; KS = soil potassium; CS/NS = TOC/TN; TOC = total organic carbon; AF = alkaline phosphatase; VA = vector angle; PS = phosphorus in soil; SE = arylsulfatase activity; CE/PE = carbon enzymes/phosphorus enzymes; VL = vector length; CaS = calcium in soil; CE/NE = carbon enzymes/nitrogen enzymes; MR = basal microbial respiration; CE = carbon enzymes; PE = phosphorus enzymes; TN = total nitrogen; CEC = cation exchange capacity; MBC = microbial biomass carbon; NS/PS = TN/PS; NE = nitrogen enzymes; MBN = microbial biomass nitrogen; NaS = sodium in soil; CS/PS = TOC/PS; MgS = magnesium in soil; NE/PE = nitrogen enzymes/phosphorus enzymes.

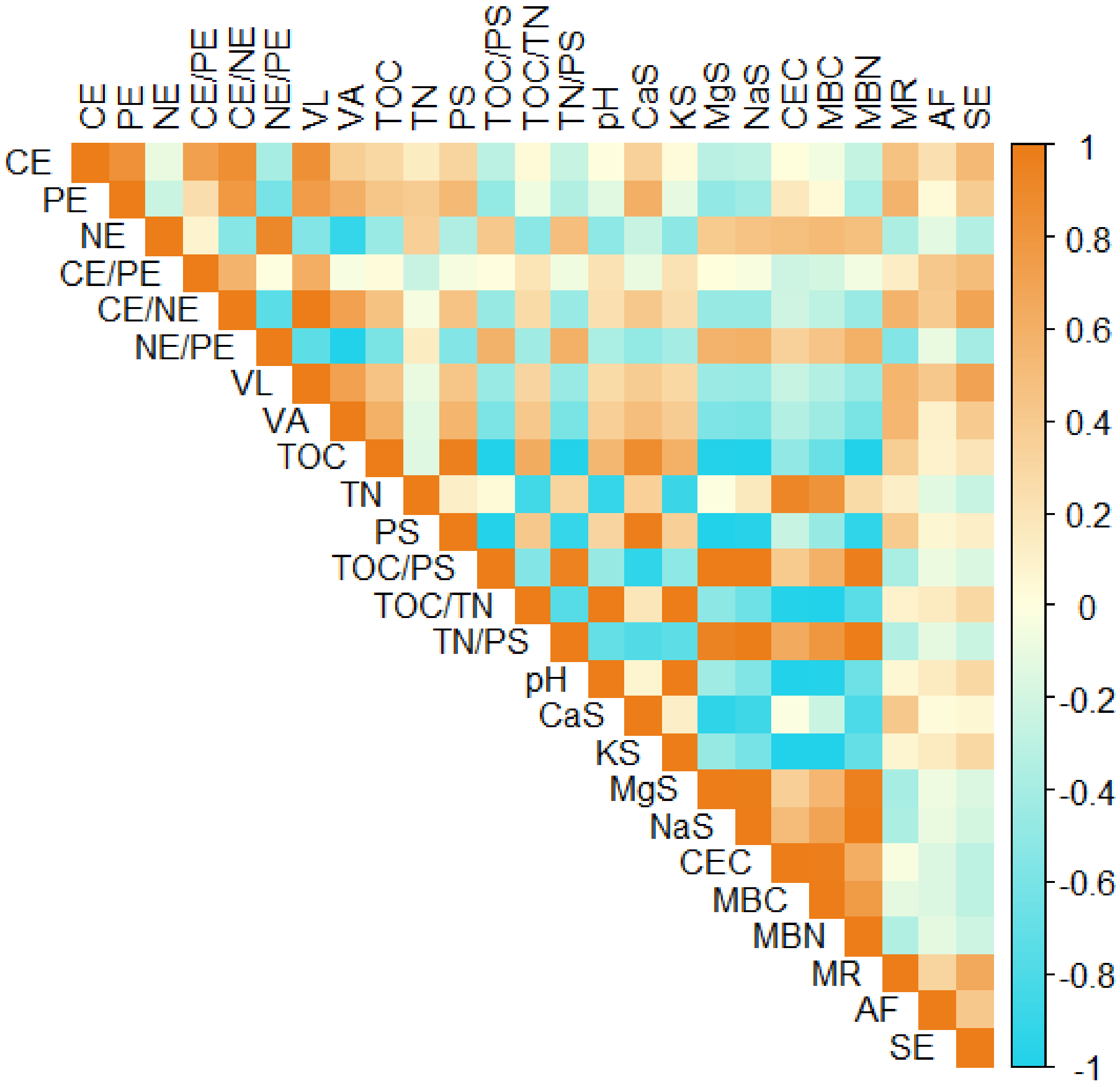

Supplementary 3 (Figure 3): Distribution evaluated for the *Agave sisalana*, *Agave* H11648 and *Agave* H400f groups between principal components (PC) 1 and 2 of the principal component analysis carried out with 25 chemical and biological soil variables of the three *Agave* species analyzed.

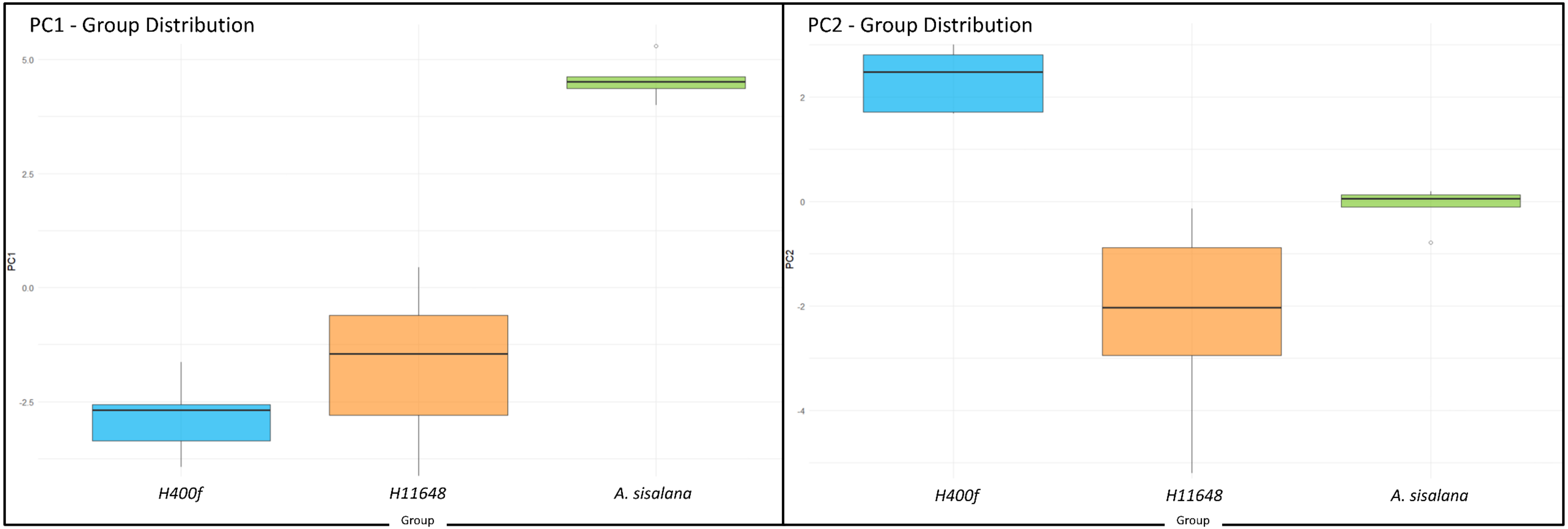

Supplementary 3 (Figure 4): Histogram obtained from the frequency of the values obtained for principal components (PC) 1 and 2 of the principal component analysis of the 25 chemical and biological variables of *Agave sisalana*, *Agave* H11648 and *Agave* H400f to check the normality of the principal components.

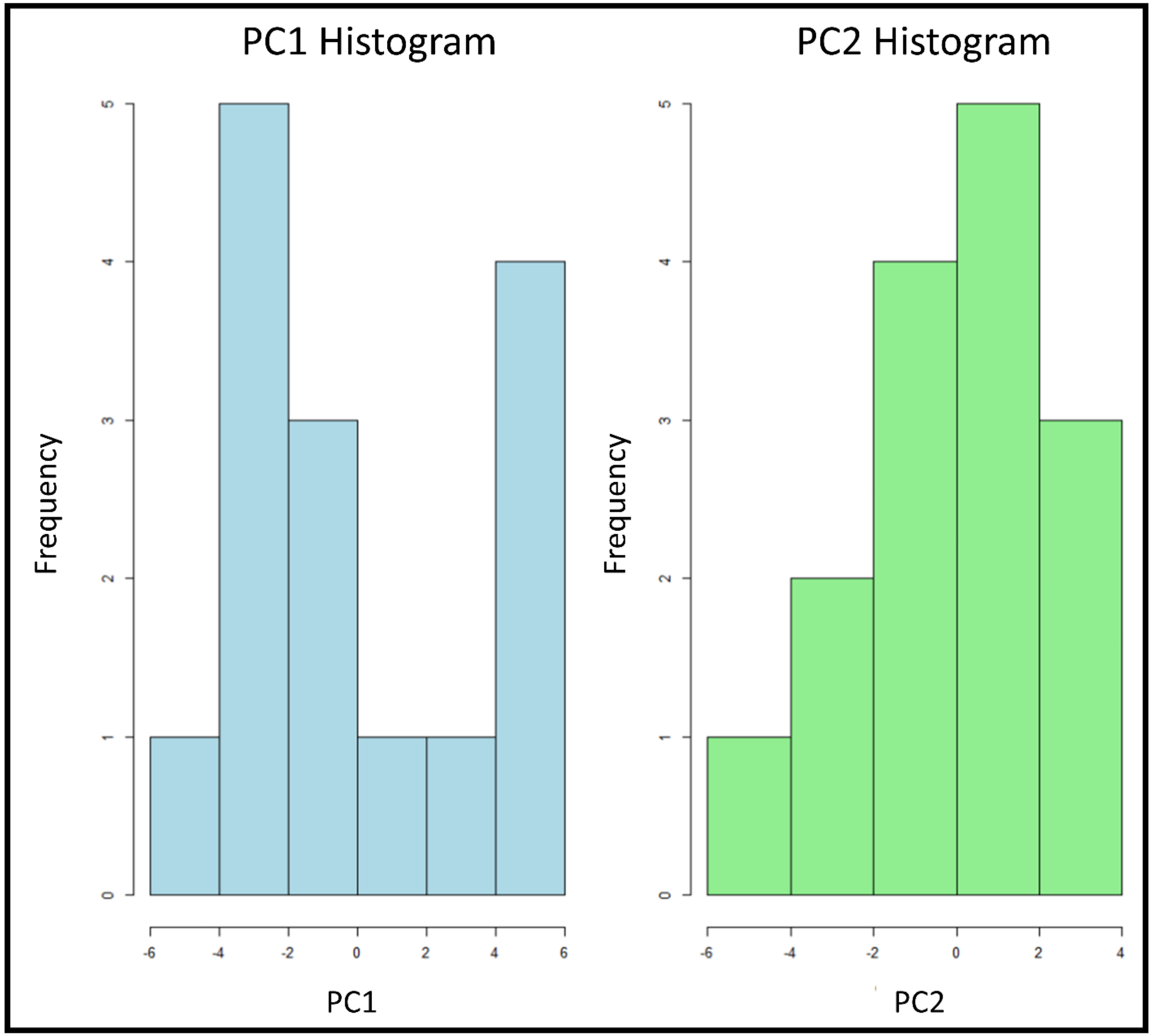

Supplementary 3 (Figure 5): Scatterplot of the *Agave sisalana*, *Agave* H11648 and *Agave* H400f groups for the principal component analysis carried out with the 25 chemical and biological soil variables of these three *Agave* species.

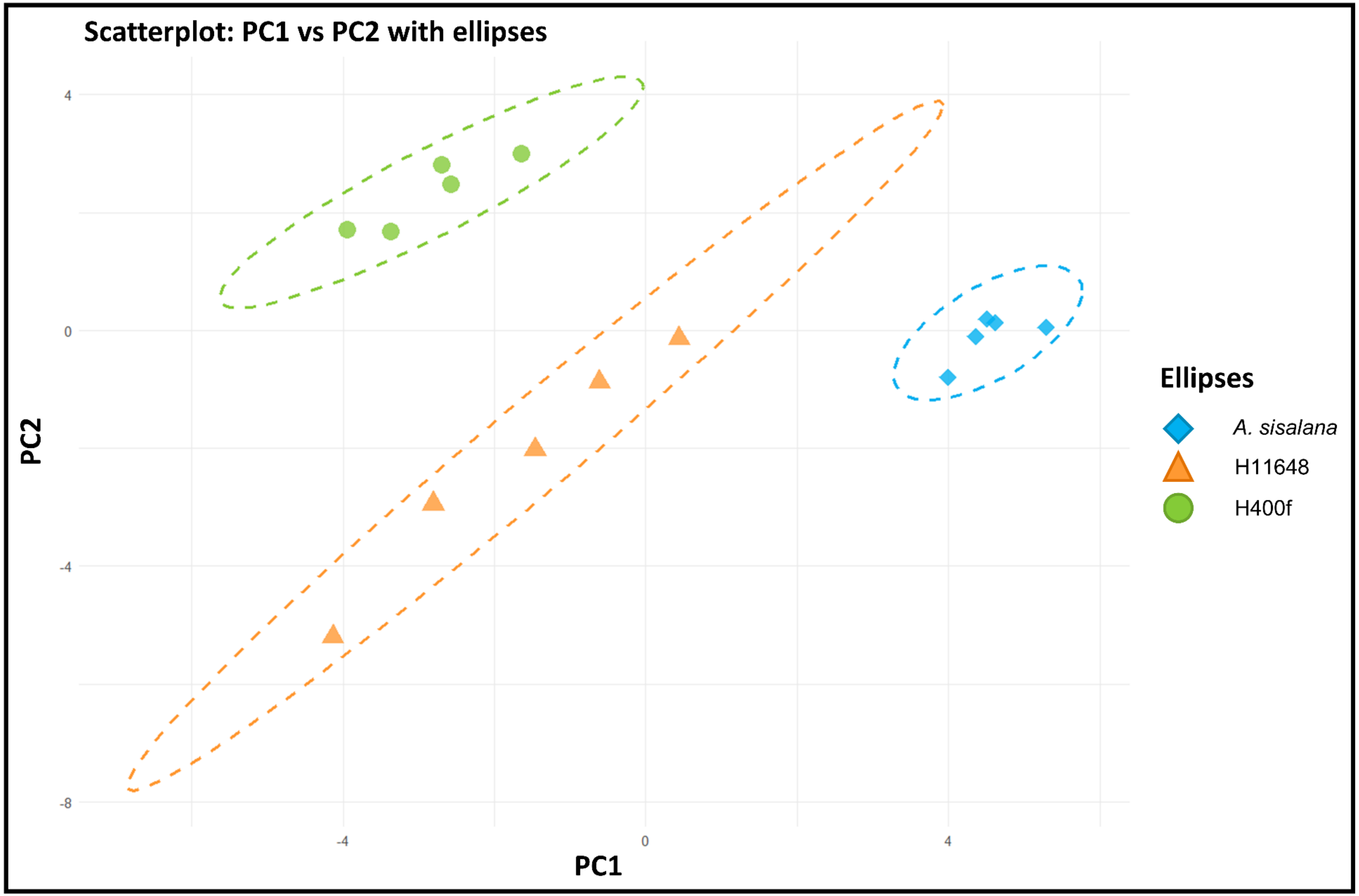

Supplementary 3 (Figure 6): Scree plot of the percentages of variance explained by the principal components (PC) obtained from the principal component analysis carried out with the 25 chemical and biological soil variables of *Agave sisalana*, *Agave* H11648 and *Agave* H400f.

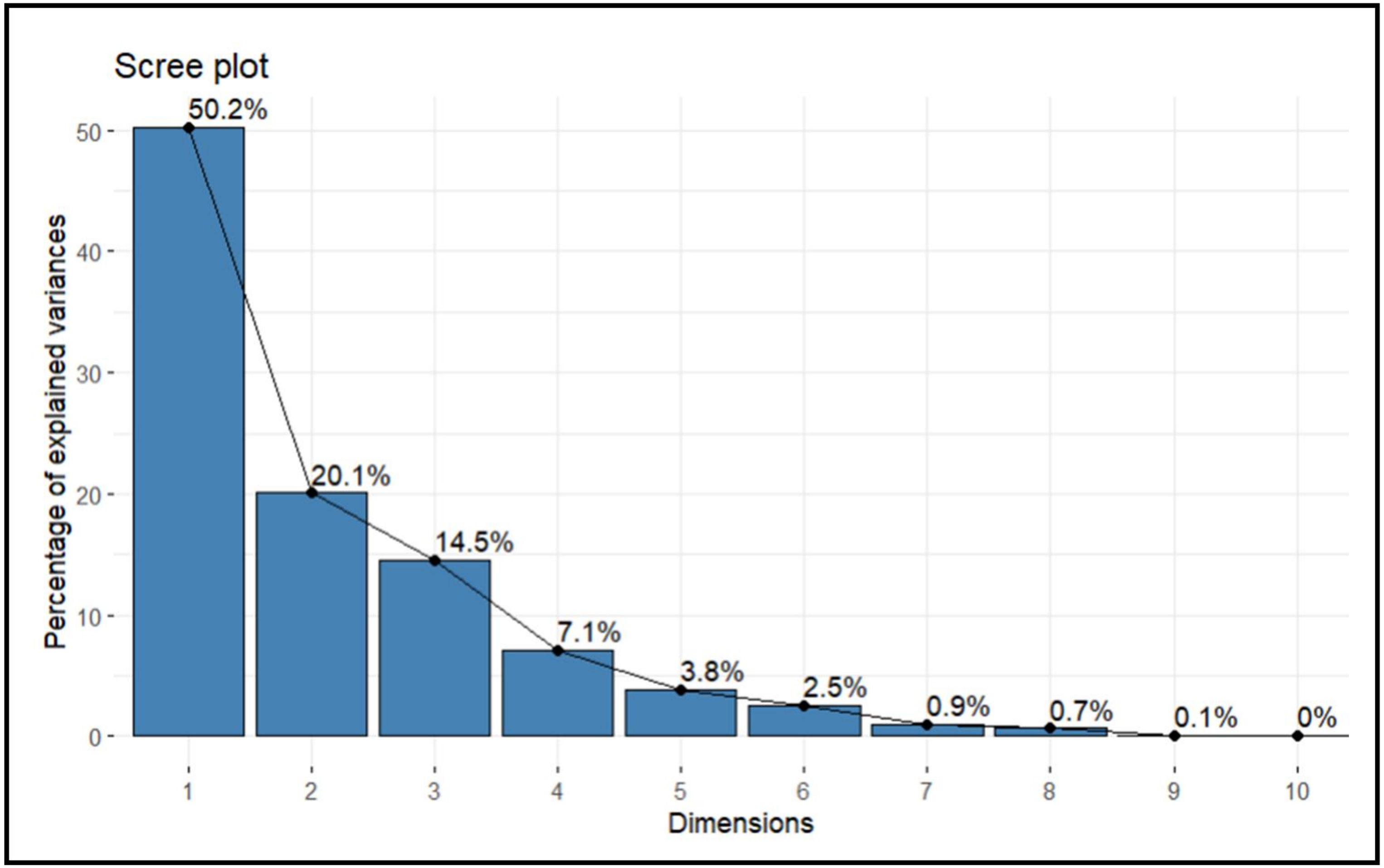

Supplementary 3 (Figure 7): Redundancy Analysis (RDA) biplot performed with the 25 response and environmental variables collected for the soil of *A. sisalana*, H11649, and H400f. Response Variables: Carbon Enzymes (CE); Phosphorus Enzymes (PE); Nitrogen Enzymes (NE); CE/PE; CE/NE; NE/PE; Vector Length (VL); Vector Angle (VA); Alkaline Phosphatase (AF); Arylsulfatase (SE); Microbial Biomass Carbon (MBC); Microbial Biomass Nitrogen (MBN); Microbial Basal Respiration (MR). Environmental Variables: Total Organic Carbon (TOC); Total Nitrogen (TN); Phosphorus (PS); TOC/PS; TOC/TN; TN/PS; Ph; Calcium (CaS); Potassium (KS); Magnesium (MgS); Sodium (NaS); Cation Exchange Capacity (CEC). Only TOC and TN were considered non-redundant.

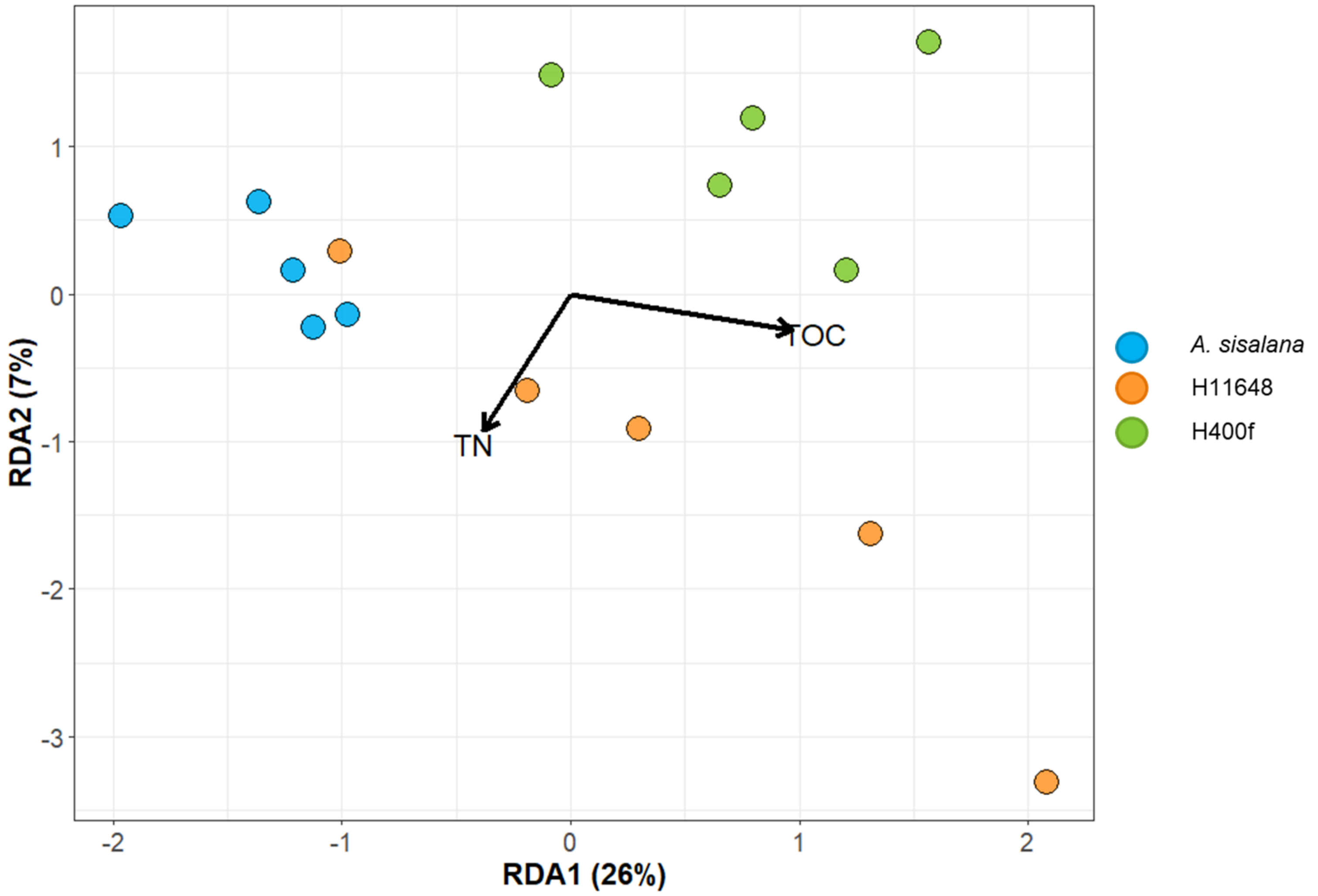

Supplementary 3 (Figure 8): PERMANOVA biplot created from the 25 variables collected for the soil of *A. sisalana*, H11648 and H400f. A = Biplot with the grouping of the samples; B = Scatter plot, with ellipses at 95% significance. Variables: Carbon Enzymes (CE); Phosphorus Enzymes (PE); Nitrogen Enzymes (NE); CE/PE; CE/NE; NE/PE; Vector Length (VL); Vector Angle (VA); Alkaline Phosphatase (AF); Arylsulfatase (SE); Microbial Biomass Carbon (MBC); Microbial Biomass Nitrogen (MBN); Microbial Basal Respiration (MR); Total Organic Carbon (TOC); Total Nitrogen (TN); Phosphorus (PS); TOC/PS; TOC/TN; TN/PS; Ph; Calcium (CaS); Potassium (KS); Magnesium (MgS); Sodium (NaS); Cation Exchange Capacity (CEC).

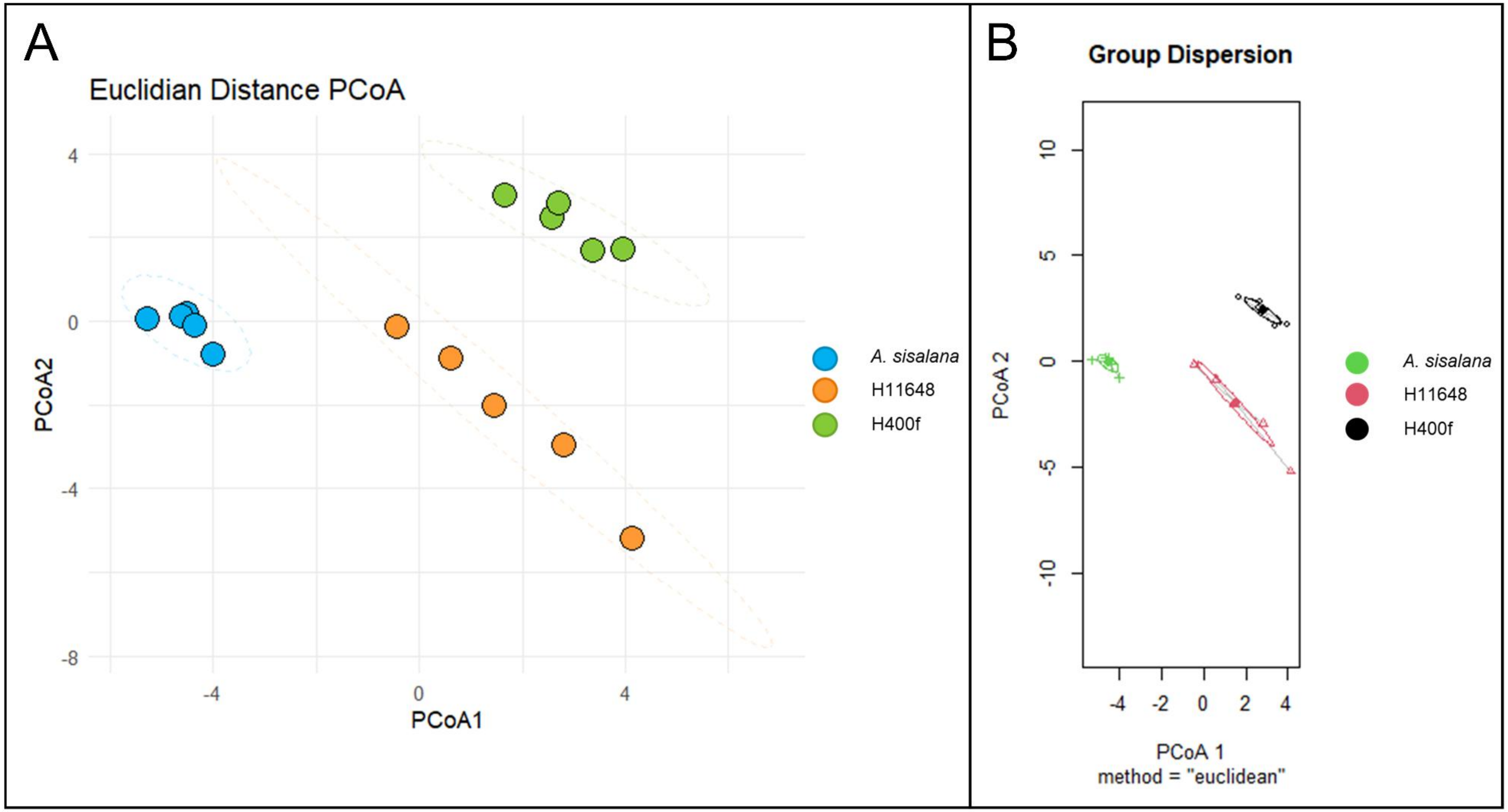

Supplement: Uncited Supplementary Material 1. [file mic-172-01681-s001.pdf]
